# Selection signatures in four German warmblood horse breeds: Tracing breeding history in the modern sport horse

**DOI:** 10.1371/journal.pone.0215913

**Published:** 2019-04-25

**Authors:** Wietje Nolte, Georg Thaller, Christa Kuehn

**Affiliations:** 1 Institute of Genome Biology, Leibniz-Institute for Farm Animal Biology, Dummerstorf, Germany; 2 Institute for Animal Breeding and Husbandry, Christian-Albrechts-University Kiel, Kiel, Germany; 3 Faculty of Agricultural and Environmental Science, University of Rostock, Rostock, Germany; Erasmus University Medical Center, NETHERLANDS

## Abstract

The study of selection signatures helps to find genomic regions that have been under selective pressure and might host genes or variants that modulate important phenotypes. Such knowledge improves our understanding of how breeding programmes have shaped the genomes of livestock. In this study, 942 stallions were included from four, exemplarily chosen, German warmblood breeds with divergent historical and recent selection focus and different crossbreeding policies: Trakehner (N = 44), Holsteiner (N = 358), Hanoverian (N = 319) and Oldenburger (N = 221). Those breeds are nowadays bred for athletic performance and aptitude for show-jumping, dressage or eventing, with a particular focus of Holsteiner on the first discipline. Blood samples were collected during the health exams of the stallion preselections before licensing and were genotyped with the Illumina EquineSNP50 BeadChip. Autosomal markers were used for a multi-method search for signals of positive selection. Analyses within and across breeds were conducted by using the integrated Haplotype Score (iHS), cross-population Extended Haplotype Homozygosity (xpEHH) and Runs of Homozygosity (ROH). Oldenburger and Hanoverian showed very similar iHS signatures, but breed specificities were detected on multiple chromosomes with the xpEHH. The Trakehner clustered as a distinct group in a principal component analysis and also showed the highest number of ROHs, which reflects their historical bottleneck. Beside breed specific differences, we found shared selection signals in an across breed iHS analysis on chromosomes 1, 4 and 7. After investigation of these iHS signals and shared ROH for potential functional candidate genes and affected pathways including enrichment analyses, we suggest that genes affecting muscle functionality (*TPM1*, *TMOD2-3*, *MYO5A*, *MYO5C*), energy metabolism and growth (*AEBP1*, *RALGAPA2*, *IGFBP1*, *IGFBP3-4*), embryonic development (*HOXB*-complex) and fertility (*THEGL*, *ZPBP1-2*, *TEX14*, *ZP1*, *SUN3* and *CFAP61*) have been targeted by selection in all breeds. Our findings also indicate selection pressure on *KITLG*, which is well-documented for influencing pigmentation.

## Introduction

Since the early onset of domestication, humans have shaped livestock species according to their purposes and current needs. Especially since the establishment of studbooks and the definition of explicit breeding goals and programmes, selection pressure has increased [1].

Regardless whether horses (*Equus caballus*) were used for warfare, transportation, farming or sports, the emphasis has first and foremost been on physical performance. Within the 20^th^ century warmblood horses have increasingly been used and bred for competitive sports disciplines such as show-jumping, dressage and eventing.

For these three disciplines, the World Breeding Federation for Sport Horses annually releases rankings for the internationally most successful studbooks. The German warmblood breeds Holsteiner, Hanoverian, Oldenburger and Trakehner have constantly belonged to the top segment in at least one discipline. In Germany, the Hanoverian and Oldenburger studbook are the two largest breeding associations in terms of the number of registered broodmares and sires, whilst the Holsteiner and Trakehner studbook rank on places 4 and 6 [2]. Taken together, the four breeds account for two thirds of the warmblood horse breeding population in Germany.

Currently, the different warmblood horse breeds in Germany essentially share selection goals regarding conformation, locomotion and aptitude for different sport disciplines. However, every breed has, in the course of time, been subjected to specific selection pressures. Thus, the four breeds Holsteiner, Trakehner, Oldenburger and Hanoverian serve as representatives of modern sport horses with divergent breed histories.

From the very start, the Trakehner breeding goal was on creating riding horses, initially for cavalry, and the breed has not undergone a change in utilization like the other three. The Trakehner also went through a severe bottleneck shortly after the Second World War when the population shrank from over 25,000 to about 1,500 breeding animals [3]. Compared with the other three breeds, Trakehner horses have been close to purebred for 250 years. Foreign sires are only seldom accepted into the studbook and generally English thoroughbreds and Arabians are used for refinement [4]. The proclaimed Trakehner breeding goal is a multitalented leisure and sport horse. The breed has a longstanding tradition in cross country riding and eventing and the breeding programme includes (optional) special performance tests for this discipline [4].

Hanoverian horses were originally bred for primary use in agriculture and secondly for military purposes. After the Second World War, the change in breeding orientation changed towards a lighter riding horse, and therefore Thoroughbreds and Trakehner were increasingly included in the breeding scheme [5].

The Oldenburger breed was primarily intended for carriage driving and favoured heavier warmblood horses [6] in the early 20^th^ century. In contrast to Hanoverian, the Oldenburger studbook remained closed and practiced pure breeding for a relatively long time and started breeding for lighter riding horses only since the 1950s [7, 8]. Since then the Oldenburger breeding goal constitutes a powerful high-performance sport horse with aptitude for all kinds of disciplines [9], analogous to the Hanoverian studbook that selects for an aptitude for show-jumping, dressage, eventing or carriage driving [10].

Nowadays, Hanoverians and Oldenburger both have a specialised breeding programme for show-jumping, although their formats differ. The Hanoverian studbook opened a specialised jumping programme in 1993 that promotes the pairing of broodmares and sires with proven suitability for this discipline [5].

In 2001, the studbook Oldenburg International was founded, which is oriented on show-jumping [11], so the original studbook can predominantly breed for dressage aptitude. Both studbooks can operate independently from one another but belong to the Oldenburger breeding association.

In contrast to Trakehner, Hanoverian and Oldenburger accept sires from a number of different warmblood horse breeds for refinement, as long as their selection criteria are met. English thoroughbreds and Arabians are also acceptable breeds for refinement.

Historically, Holsteiner horses have been primarily used as draught horses in agriculture and transportation and have been rarely selected for riding. In the middle of the 20^th^ century the breeding goal shifted from a use in agriculture to sports and today they have an explicit focus on show-jumping. To refine the breed, English thoroughbreds, Arabians and French warmblood horses may be accepted and in case of special aptitude for jumping also sires from other warmblood breeds [12].

Particularly in the Holsteiner and Hanoverian breed the intensive use of a few sires in the 20^th^ century possibly gave rise to popular sire effects [13, 14].

Considering the clearly sports-oriented current breeding programmes of all four studbooks in question, we hypothesized that selection pressure on genes relevant for athleticism and suitability for one of the major disciplines (show-jumping, dressage, eventing) should be reflected on a molecular genetic level. Sorbolini et al. [15] demonstrated in cattle that breeds—in spite of similar phenotypes and breeding goals—still have divergent selection signatures due to historic differences. We expected to see a similar phenomenon in sport horse breeds, potentially due to historically divergent main breeding goals.

When an advantageous allele is favoured in the selection process it usually segregates together with neighbouring, so-called hitchhiking alleles. Selective sweeps occur when such genomic segments spread over generations throughout the population due to artificial or natural selection, consequentially bringing about a reduction of genetic variation in those parts of the genome [16]. The study of selective sweeps can therefore give insights into the historical development of populations and is valuable for the unravelling of the functional, genetic background leading to phenotypic variation [17].

Many approaches based on intra- and inter-population statistics have been successfully applied to humans [18, 19] as well as domesticated animals [20].

Runs of Homozygosity (ROH) refer to continuously homozygous segments in the genome and have already led to the identification of genomic regions and putative candidate genes that are under selection in domestic animals [21–23]. In Haflinger horses this method has also been applied to assess breed history and development [24, 25]. A previous ROH study comprising divergent horse breeds, which have been subjected to very different degrees of selection pressure, suggested genes to be targeted that influence metabolic, developmental and neurological processes as well as pigmentation and fertility [26].

The integrated Haplotype Score (iHS) and the cross-population Extended Haplotype Homozygosity (xpEHH) are two other methods for the detection of selection signatures based on haplotype information. The iHS is particularly suitable to detect incomplete sweeps within populations, whereas the xpEHH can better be used to detect (nearly) complete sweeps, i.e. sites that are still polymorphic in one population but are fixed in another [16]. Both approaches have been applied in different horse breeds such as Asian [27] and Shetland ponies [28], where growth, height, feed efficiency and fat deposition related genes appeared to have been under selective pressure. Furthermore, racing performance and locomotion have been targeted in gaited breeds and Quarter Horses [29].

In thoroughbred horses, the search for selection signatures revealed regions that harbour genes associated with muscle strength, energy pathways, insulin signalling, and lipid metabolism, which reflects their breeding for racing performance [30]. The selection for athletic performance in Quarter Horse populations also appears to have put selective pressure on metabolism, next to skeletal muscle development and the nervous central system [31].

Clearly, selection for athletic performance has left traces in the genome of different horse breeds and we hypothesized that similar developments have occurred in the European warmblood horse.

The aim of this study was to identify genomic regions under positive selection within and across warmblood horse breeds. We further sought to elucidate whether differences in breed histories can be detected through selection signatures. Based on the detected selection signatures we intended to present candidate physiological processes and putative candidate genes for phenotypic traits that have been of special interest to breeders.

## Material and methods

### Sample data

A total of 942 stallions (*Equus caballus*) from the four warmblood horse breeds Trakehner (N = 44), Holsteiner (N = 358), Oldenburger (N = 221) and Hanoverian (N = 319) were sampled during the health check of the stallion preselections before licensing and represent the birth years 2002–2006 (Table 1). The study made exclusively use of existing data collected for a previous project and no specific sampling was conducted. Blood samples were taken by licensed veterinarians as part of the mandatory health and parentage check in the licensing procedure for stallions in Germany. Since the health and parentage checks are legally mandatory for stallion licensing no ethical approval procedure was necessary. All animals had passed an initial first inspection, but the sampling was independent of passing the health check subsequent to the initial inspection and of the final licensing decision. To pass the first inspection, stallions need to be free of deficiencies in conformation and movement and need to have a pedigree that fits the individual studbook requirements. Stallions presented for preselection are generally 2.5 to 3 years of age. The sample includes stallions that stem from show-jumping and dressage lines as well as stallions with a presumed aptitude for eventing. Stratification due to breeding lines for show-jumping or dressage aptitude can be neglected [32]. The EDTA-stabilized blood samples were used as sources of DNA for genotyping on the EquineSNP50 BeadChip (Illumina Inc., CA). Filter options for SNPs were set to MAF <0.01, call frequency <0.9 and p(χ^2^) <0.00001 for Hardy-Weinberg-Equilibrium in the Illumina Genome Studio used for the analyses. After filtering, 48,410 SNPs (overall genotype call rate of 99.879 percent) on 31 autosomal chromosomes remained for statistical analysis. Allosomes were not considered, because no Y chromosome data were available and allosomes would not enable homozygosity based analyses in male individuals.

**Table 1:** Distribution of stallions included in the study by year of birth and breed.

birth	Trakehner	Holsteiner	Hanoverian	Oldenburger
2002	1	0	0	3
2003	0	86	79	60
2004	19	90	90	68
2005	16	90	70	65
2006	8	92	80	25

### Data processing and statistical analysis

For the detection of selection signatures three methods were applied: ROH, iHS and xpEHH. Before statistical analyses, haplotypes were derived and missing genotype calls were imputed chromosome wise for all samples together across breeds in Beagle 4.0 [33] while neglecting pedigree information. Given the very high average call rate (>99.9 percent), the proportion of imputed genotypes in the final dataset was extremely low (0.121 percent). Since the Trakehner sample comprised less than 50 animals, which is usually considered a lower limit for quality imputation, we performed the imputation across all breeds together.

To capture population structure, a principal component analysis (PCA) of the genotype dataset was done with the software Genome-wide Complex Trait Analysis (GCTA), version 1.91.7beta [34, 35]. A genomic relationship matrix was built from the genotype information and used to calculate the first 20 eigenvectors and all eigenvalues.

### Runs of Homozygosity

ROH and their clusters, i.e. homozygous segments shared by multiple individuals, were analysed chromosome-wise using the SNP & Variation Suite v.8.8.1 [36]. ROH-clusters were analysed within and across breeds. The across and within breed clusters of ROHs were defined by segments shared by at least a third of the individuals. The distance minimum was set to 500kb and 15 SNPs and no missing or heterozygous SNPs were accepted. The lower density limit was set to 1 SNP per 100kb and we allowed for a maximum gap distance of 1,000kb [37].

### Haplotype-based analyses

Voight et al. [19] introduced iHS as a modification of the Extended Haplotype Homozygosity (EHH) previously developed by Sabeti and colleagues [38]. The EHH captures the decay of homozygosity with increasing distance from a core allele. An allele under strong selection will usually be embedded in an unexpectedly long homozygous haplotype which is in contrast to the unfavoured allele. This difference between ancestral and derived alleles is described as the iHS and equates the standardised quotient of the integral under the EHH curves of the ancestral and derived allele.

A large positive value hence indicates that an ancestral allele is under positive selection and has increased in frequency but has not yet obtained fixation. A large negative value results from selection for the new, derived allele [16]. The iHS-computations were done per chromosome for individuals within and across breeds. By applying the iHS across all breeds, we aimed to pick up selection signals that affect the group as a whole. The pooling of all four breeds together treats them as the sport horse population as a whole and provides a more comprehensive perspective.

When a selected allele has reached fixation within one population but is still polymorphic in another, the xpEHH as described by Sabeti et al. [18] has a very high statistical power to detect such differences between populations. Hence, it successfully discovers complete selective sweeps within a specific breed [16]. The xpEHH is derived from pairwise breed comparisons. We compared each breed individually (“case population”) to the total of the other three breeds combined (“control population”).

For iHS and xpEHH, information on the allele status is required, defining alleles as ancestral and derived. SNP data from a domestic ass (*Equus asinus*), serving as outgroup, were used to deduce the putative allele status (http://geogenetics.ku.dk/publications/middle-pleistocene-omics, accessed 13 July 2016).

For comparison with the general caballoid state, the reference genome EquCab2.0 [39] was used. This follows the assumption that the donkey still possesses ancestral alleles while new “derived” alleles have emerged through mutation events in the modern horse and have then increased in frequency through domestication or breed formation. This approach is commonly used in in selection signature studies, e.g. chimps are used as outgroup for humans and bison, yak or buffalo for cattle [19, 40].

A total of 48,410 SNPs were entered in the iHS and xpEHH analyses. Calculations of both iHS and xpEHH were executed in R Statistical Software using the tailored package REHH 2.0.0 [41] with default options. A linkage disequilibrium evaluation (r^2^ ≥ 0.8), based on phased and imputed data and executed in Haploview 4.2 [42], resulted in 7,739 tag SNPs across all autosomes. We therefore assumed a conservative significance threshold of p = 0.0001 (-log_10_(p-value) = 4.0) equivalent to 10,000 independent tests to account for multiple testing.

### Screening for candidate genes

For functional analysis, regions covering selection signatures were scanned for annotated genes in the equine reference assembly EquCab2.0 using the online tool Biomart from Ensembl (https://www.ensembl.org/biomart/martview, accessed April 2018, Ensembl release v92). Breed overlapping iHS-signatures were checked 1Mb up- and downstream from the significant SNP. With regard to ROH-clusters, the positional resolution of the beadchip is comparatively low, and in order to avoid too many false positives the scanning for annotated genes was done conservatively within the margins of each particular ROH-stretch.

For the functional interpretation of the signatures, the assumption was made that signals were due to artificial or natural selection pressures and not due to demography.

To identify putative candidate genes under selection pressure we took into account (A) which Quantitative Trait Loci (QTL) fell into selection signatures, (B) which genes have a potential functional link to the pronounced breeding goals of these horse breeds, (C) which important biological pathways were identified through an enrichment analysis, and (D) which genes have been reported in relevant literature.

- For results from the across-breed iHS and ROH as well as the xpEHH, we checked for intersection of these selection signatures with known QTL in horses downloaded from the animalgenome.org database (https://www.animalgenome.org/ cgi-bin/QTLdb/EC/summary, accessed February 2019, release 37). The intersection of QTL regions and selection signatures was done with bedtools intersect [43], filtering for a complete overlap. Analogously to the scanning for annotated genes (see above), the selection signatures of iHS and xpEHH were extended by 1MB up- and downstream for this analysis, while no margin adjustment was done for the ROH.
- We paid special attention to genes related to growth, fertility, conformation, pigmentation, metabolism, athletic performance and locomotion since these aspects are part of the more detailed selection criteria in the statutes of the studbooks.
- To see which biological pathways might have been targeted across breeds, we used the list of annotated genes within ROH and iHS selection signatures for an enrichment analysis in the functional annotation tool DAVID 6.8 [44, 45] (https://david.ncifcrf.gov/, accessed 14 Feburary 2019). The gene lists were analysed for the species *Equus caballus* against the matching background. The Benjamini and Hochberg [46] test was used to correct for multiple testing.
- We thoroughly crosschecked with literature which genes have been found or suggested as targets in previous selection signature or association studies in horses and other domestic species. For instance a PubMed search in the National Center for Biotechnology Information (NCBI) database yielded 26 hits for the keywords “horse selection signatures” and 43 hits for “domestic animals selection signatures”. These and other topic related publications, such as the studies fed to the HorseQTLdb (https://www.animalgenome.org), were considered for the determination of candidate genes.

## Results

### Principal component analysis

**Fig 1:**
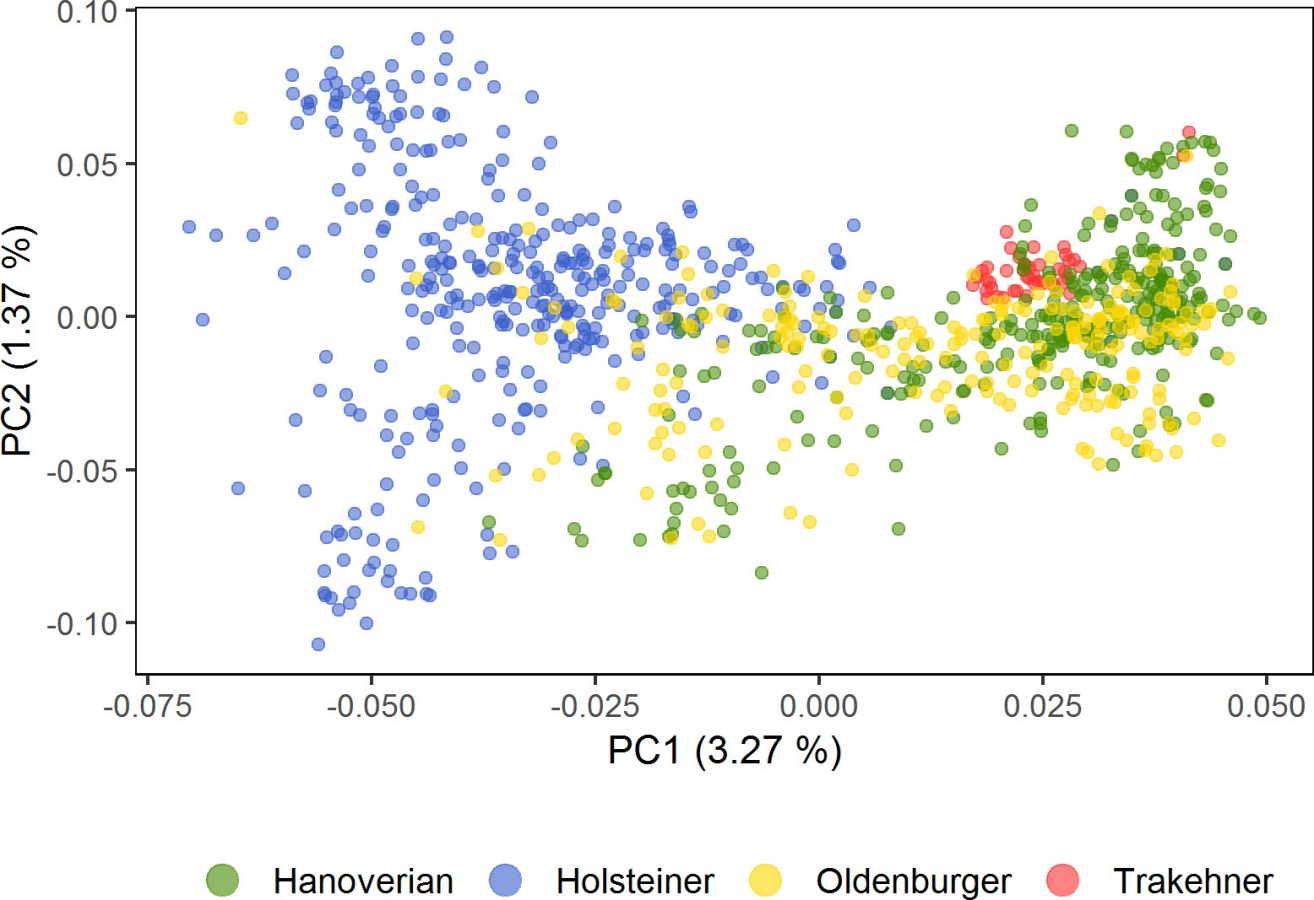
Principal Component Analysis (PCA) based on genotype data for four German warmblood horse breeds. Based on a genomic relationship matrix, eigenvalues were calculated and the first two components used for a colour-coded clustering of the breeds Hanoverian (N = 319), Holsteiner (N = 358), Oldenburger (N = 221), and Trakehner (N = 44).

A plotting of the first two principal components of the genotype data resulted in a tentative separation of the dataset into the four breeds (Fig 1). The Trakehner cohort forms a distinct subgroup and nests next to Oldenburger and Hanoverian, which mostly overlap. Holsteiner cluster more separately from the other three breeds.

### Selection signatures intersecting with QTL

When considering across breed iHS and xpEHH selection signatures (both ±1Mb) and ROH shared by at least a third of all samples, these overlap with 44 QTL known in horses. Out of the equine 2,023 QTL listed in the animal QTL database, 1,975 are on autosomes and have a physical position in base pairs. The 44 QTL we found to fall within selection signatures belong to a total of 12 different traits (Table 2). Since some traits are represented with a much higher number of QTL in the database than others, we set the number of overlapped QTL in relation to the known total. Four traits were identified for which over 10 percent of the listed QTL fall into selection signatures: cannon bone circumference, coat texture, hair density and sperm count.

**Table 2:** Overlap of known QTL with selection signatures in four warmblood horse breeds.

trait	known QTL^1^	overlap selection signatures (iHS^2^, ROH^3^, xpEHH^4^)	percentage of known QTL
**alternate gaits**	75	1	1.3
**body weight**	46	1	2.2
**cannon bone circumference**	8	1	12.5
**coat texture**	1	1	100.0
**guttural pouch tympany**	263	9	3.4
**hair density**	7	5	71.4
**height of withers**	529	16	3.0
**insect bite hypersensitivity**	84	3	3.6
**osteochondrosis dissecans**	115	1	0.9
**racing ability**	86	3	3.5
**sperm count**	4	1	25.0
**white markings**	78	2	2.6

^1^ QTL with physical coordinates in base pairs available

^2^ integrated Haplotype Score selection signature ±1Mb in across breed analysis

^3^ Run of Homozygosity shared by at least a third of all individuals

^4^ cross-population Extended haplotype Homozygosity selection signature ±1Mb

### Runs of Homozygosity

The search for ROH clusters, i.e. homozygous segments shared by multiple individuals, yielded selection signals within and across breeds. The across-breed approach (N = 942) revealed 37 such signatures on 16 different chromosomes, reaching a maximal length of 47 SNPs or 2,294,884bp (Table 3). Up to 43 percent (N = 404) of the sampled horses shared a particular ROH-segment.

**Table 3:** Runs of Homozygosity (ROH) shared by at least 33 percent of all individuals (N = 942) across four warmblood horse breeds with candidate genes for positive selection.

Chr	position (bp)	length of ROH (bp)	SNPs in ROH	samples (%) sharing the entire ROH	annotated genes in ROH	candidate genes	QTL trait (number of QTL) ^1^ [47]
1	684,531–1,346,011	661,480	20	33	5		
	21,937,435–22,763,212	825,777	20	33	-		
2	96,475,507–97,254,688	779,181	16	33	1		
	100,347,967–100,961,025	613,058	19	35	5		
3	19,390,495–20,068,977	678,482	20	35	14		
	22,774,232–23,723,805	949,573	24	34	19		
	39,317,467–40,650,253	1,332,786	20	34	24		white^2^ (1), tympany^3^ (6)
	75,797,341–76,393,636	596,295	19	34	13	THEGL	white (1), height^4^ (2)
	118,524,879–119,456,949	932,070	38	37	18	MYL5	
4	15,120,680–17,415,564	2,294,884	40	35	24	IGFBP1, IGFBP3	
	19,079,166–20,235,933	1,156,767	19	40	10	SPATA48, ZPBP	
	52,587,338–53,148,501	561,163	22	36	4		
5	41,546,213–42,335,441	789,228	24	33	41		
	55,293,804–56,274,553	980,749	28	34	12	WNT2B	
6	29,004,794–30,308,246	1,303,452	28	34	15	WNT5B	cannon ^5^(1)
	34,084,026–35,230,778	1,146,752	25	35	51		
	41,218,272–42,713,648	1,495,376	25	37	18		
7	36,663,831–37,338,232	674,401	23	33	6		
	39,405,488–41,489,510	2,084,022	47	34	7		
8	22,113,249–23,719,695	1,606,446	32	34	43		height (1)
	36,329,323–37,420,192	1,090,869	20	34	12		height (4)
9	43,718,150–44,540,142	821,992	20	38	13		
11	21,692,258–22,655,280	963,022	19	33	35	KRT complex, IGFBP4, ZPBP2	hair ^6^(3), coat^7^ (1),
	24,038,863–24,990,015	951,152	16	38	27	IGF2BP1 HOXB complex	hair (1)
	26,909,643–27,819,093	909,450	33	37	4		
	32,322,500–33,580,784	1,258,284	29	35	39	TEX14	insect^8^ (2)
15	44,286,531–45,105,390	818,859	16	38	16		
	67,528,191–68,378,301	850,110	18	37	7		
17	20,690,428–22,444,884	1,754,456	28	33	22		racing ^9^ (1)
18	48,042,468–49,758,616	1,716,148	39	36	23	NOSTRIN, MYO3B	racing (1)
	58,962,761–59,847,420	884,659	22	34	8		weight^10^ (1)
22	4,359,148–4,944,734	585,586	15	35	5	RALGAPA2 CFAP61	
	15,520,818–16,561,219	1,040,401	18	36	6	BMP2	
	26,103,882–26,914,845	810,963	18	34	20	GDF5, SPAG4,	
25	26,318,531–26,942,120	623,589	18	43	33	*	
28	14,158,917–15,080,406	921,489	19	34	7	KITLG	
	45,594,055–46,121,975	527,920	17	34	29		

* suspected copy number variation

^1^ selection signature overlaps with QTL position, data downloaded from AnimalGenome.ORG animal QTL database (accessed 4 February 2019)

^2^ white markings

^3^ guttural pouch tympany

^4^ height of withers

^5^ cannon bone circumference

^6^ hair density

^7^ coat texture

^8^ insect bite hypersensitivity

^9^ racing ability

^10^ body weight

Breed-specific analyses detected a plethora of 149 ROH in Trakehner horses, while the other breeds had comparatively lower numbers. We found 58 ROH in Holsteiner, 39 in Hanoverian and 38 in Oldenburger (S1 Table).

### Determination of allele status

For the donkey, 46,747 out of the equine 48,410 SNPs could be identified after alignment to the equine reference genome EquCab2.0 and assigned an allele status: derived or ancestral. The donkey was homozygous for 46.6 percent of the caballoid alternative alleles and for 53.0 percent of the caballoid reference alleles. Alleles where the donkey was homozygous were treated as ancestral and the opposite alleles were categorised as the new, derived alleles. For 0.4 percent (173 SNPs) of the SNPs the donkey was heterozygous and the reference allele of the horse was then assumed to be the ancient one. The remaining 1,663 of the 48,410 SNPs were randomly assigned to either of the two allele status categories. We did not leave them out of subsequent analyses, because we searched for selection events and not selection direction, meaning that we focussed on if and where selection has occurred and not which allele was favoured over its alternative counterpart.

### Integrated Haplotype Score

**Fig 2:**
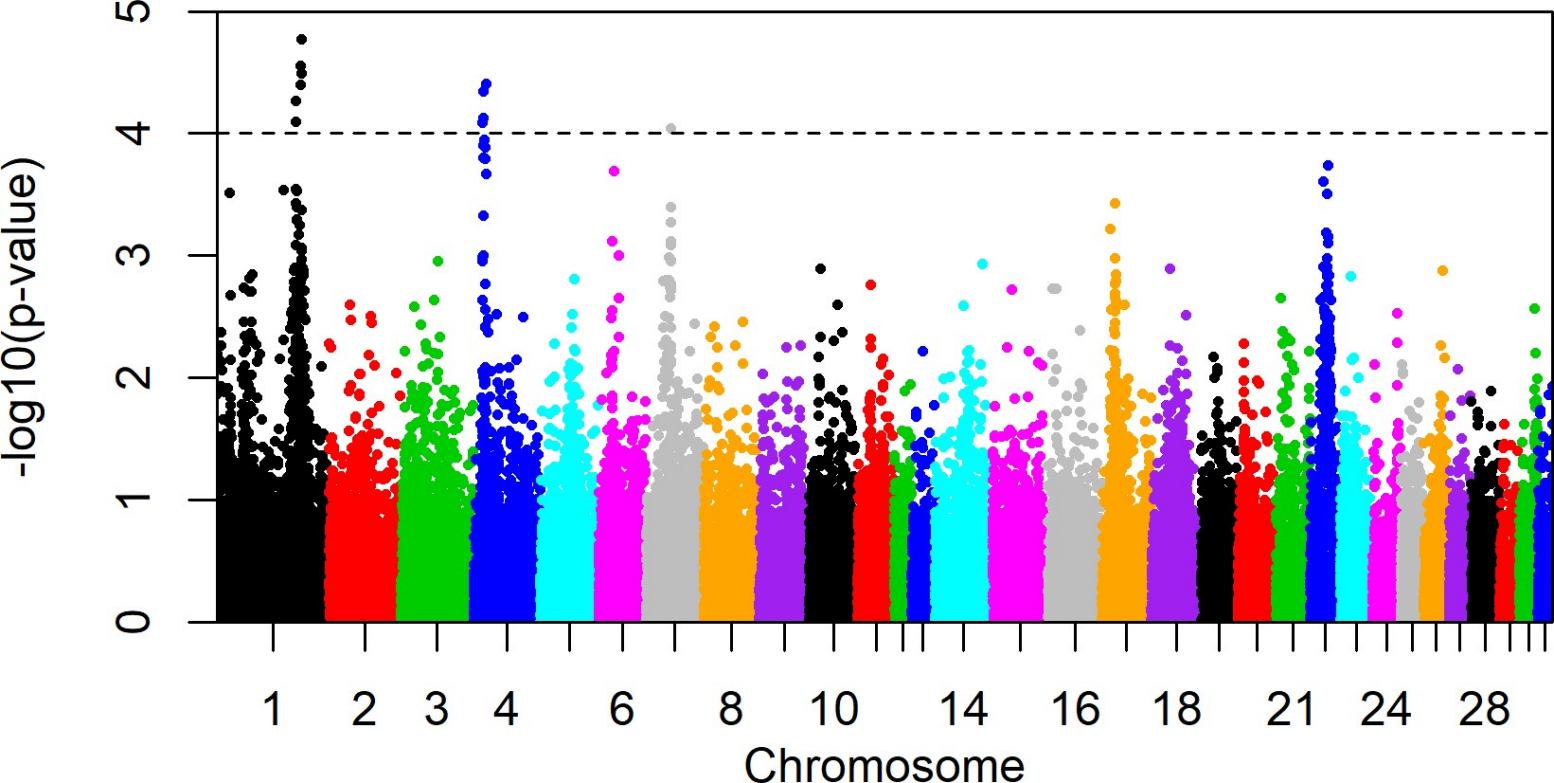
Genome-wide integrated Haplotype Score (iHS) plot across four warmblood horse breeds. Genome-wide iHS plot for all individuals (N = 942) across the four breeds Trakehner, Holsteiner, Hanoverian and Oldenburger with a significance threshold of -log_10_(p) ≥ 4.0.

**Fig 3:**
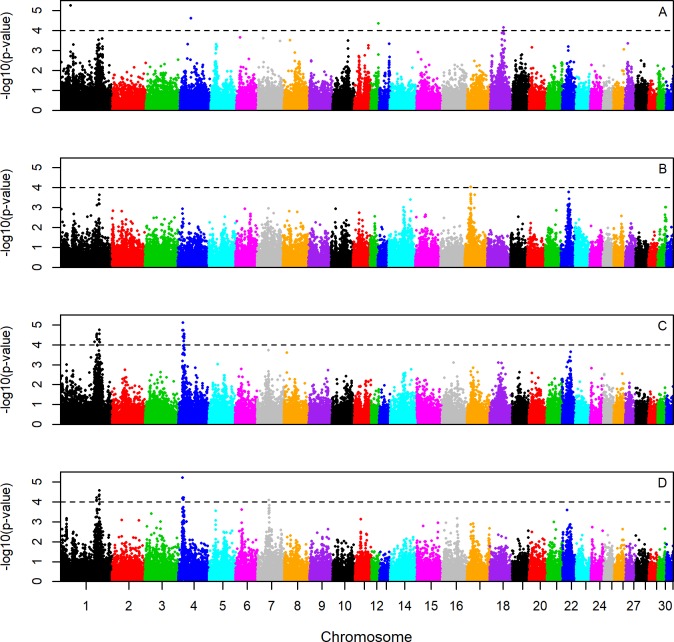
Breed-specific genome-wide integrated Haplotype Score (iHS) plots within four warmblood horse breeds. Genome-wide breed-specific iHS plots for Trakehner (A), Holsteiner (B), Hanoverian (C) and Oldenburger (D) with a significance threshold of -log_10_(p) ≥ 4.0.

In the across-breed analyses, significant signatures (-log_10_(p-value) ≥ 4.0) were found in the following regions: ECA 1 (128.78–128.83Mb and 137.76–139.27Mb), ECA 4 (13.97Mb, 16.09Mb, 17.46Mb and 20.66Mb) and ECA 7 (39.67Mb) (Fig 2, Table 4). Markers exhibiting significant iH-Scores on ECA 1 and 4 were summarized into three clusters due to their close physical localization. Breed specific analyses (Fig 3, S2 Table) revealed signatures specific to one breed or shared by more than one breed. Hanoverian and Oldenburger were characterized by a very similar signature pattern and shared signals on ECA 1 (128.78–128.83Mb and 138.48–139.26Mb) and 4 (13.97Mb, 16.09Mb, 17.45–17.50Mb and 18.00–18.45Mb). In contrast, significant signals on ECA 7 (39.67Mb) were seen in Hanoverian, while a peak on ECA 17 (23.17Mb) was found in Holsteiner and signals on ECA 1 (35.77Mb), 4 (39.14Mb), 12 (29.75Mb) and 18 (49.76Mb) were detected in Trakehner.

**Table 4:** Integrated Haplotype Score (iHS) signals across four warmblood horse breeds with a significance threshold of -log_10_(p-value) ≥ 4.0.

Chr	SNP position (bp)	iHS	-log_10_(p-value)	Extended interval of iHS-signals ± 1Mb	annotated genes at iHS-signal ± 1Mb	candidate genes	QTL trait (number of QTL) ^1^[47]
1	128,778,389	-3.94	4.1	127,778,389–129,829,558	26	TPM1, TLN2	OCD^2^ (1)
	128,829,558	-4.04	4.26				
1	137,759,895	4.19	4.55	136,759,895–140,266,776	40	MYO5A, TMOD2, TMOD3, MYO5C	
	138,481,053	4.11	4.4				
	139,162,818	4.3	4.77				
	139,266,776	4.16	4.5				
4	13,965,265	3.94	4.09	12,965,265–21,661,704	80	AEBP1, GCK, DBNL, ZPBP, SUN3, SPATA48, IGFBP1 & 3, MYO1G, MYL7	
	16,091,738	3.96	4.13				
	16,091,813	3.96	4.13				
	17,455,561	4.08	4.34				
	20,661,704	-4.11	4.41				
7	39,673,370	3.91	4.04	38,673,370–40,673,370	12	ST14, SNX19	

^1^ selection signature overlaps with QTL position, data downloaded from https://www.animalgenome.org/cgi-bin/QTLdb/index (accessed 4 February 2019)

^2^ osteochondrosis dissecans

### Cross-population Extended Haplotype Homozygosity

Analogous to the iHS-analyses, SNPs with a -log_10_(p-value) ≥ 4.0 were considered to be significant. We compared each breed (“case population”) to the other three breeds together (“control population”).

**Fig 4:**
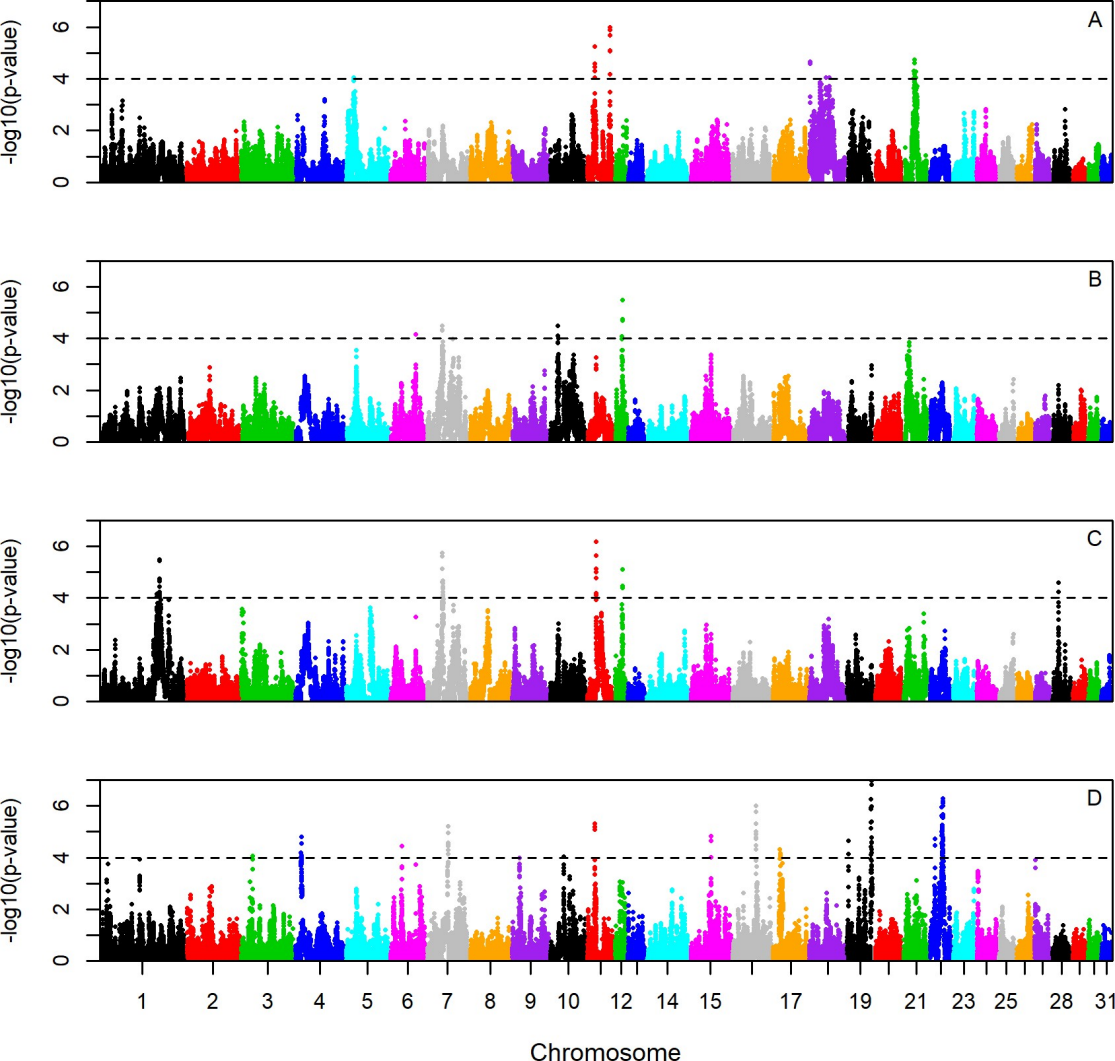
Cross-population Extended Haplotype Homozygosity (xpEHH) plot for four warmblood horse breeds. Comparison of one breed with the three others together as control for Trakehner (A), Holsteiner (B), Hanoverian (C)and Oldenburger (D) with a significance threshold of -log_10_(p) ≥ 4.0.

**Fig 5:**
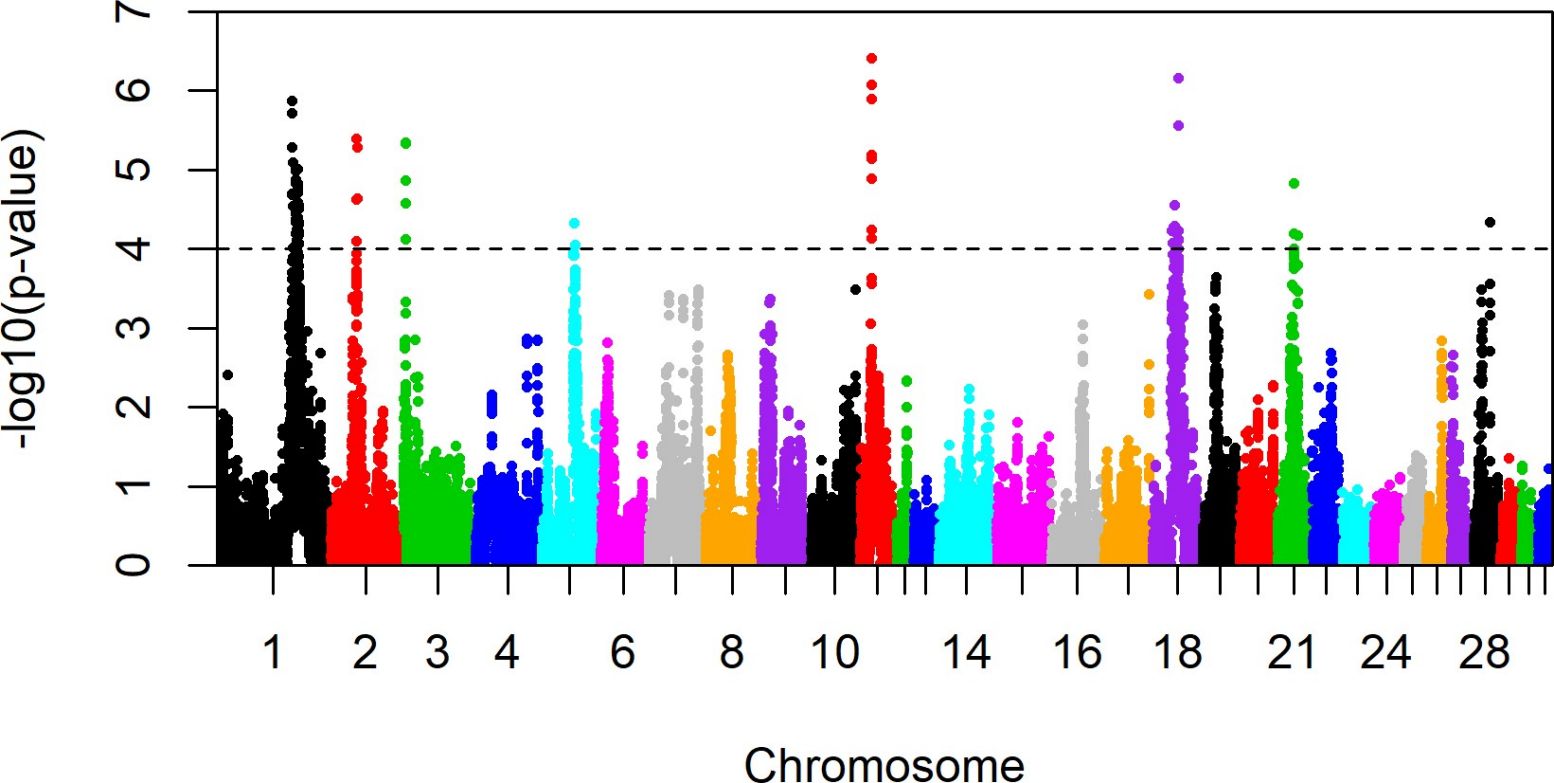
Cross-population Extended Haplotype Homozygosity (xpEHH) plot for Hanoverian versus Oldenburger breed. Comparison of the two breeds Hanoverian and Oldenburger. Significance threshold -log_10_(p) ≥ 4.0.

Trakehner exhibited significant breed-specific selection signatures on 4 different chromosomes, Holsteiner also on 4, and Hanoverian on 5 (Fig 4, Table 5). The Oldenburger breed showed numerous significant signals on 12 different chromosomes with the highest values on ECA 19 (52.3–53.9Mb). Despite similar iHS signals for Oldenburger and Hanoverian, those two breeds showed many differences when directly compared (Fig 5).

**Table 5:** Cross-population Extended Haplotype Homozygosity (xpEHH)—Comparison of one breed with the other three with a significance threshold of -log_10_(p) ≥ 4.0.

breed	Chr	position (bp)	annotated genes at xpEHH-signal ± 1Mb	candidate genes	QTL trait (number of QTL) ^5^[47]
**TRAK**^**1**^	5	18,899,521	19		
	11	17,779,157–17,946,985	49	SPATA32, WNT3, MYL4	height^6^ (1)
		51,904,224–51,910,738	39	MYH8, MYH13	
	18	2,187,680–2,187,801	17	MYO7B	height (1)
		37,572,919	16		
		44,283,029	9	GRB14	
	21	25,682,342–26,310,700	27		
		29,377,724	22		
**HOL**^**2**^	6	56,927,881	16		
	7	36,118,379–36,156,655	9		height (1)
	10	19,957,969–20,761,246	131	MYH14, MYBPC2	height (2)
	12	20,105,998–20,154,683	63	ZP1	insect^7^ (1), height (2)
**HAN**^**3**^	1	123,021,257–123,023,963	11		
		128,680,555–128,914,146	28	TLN2, TPM1	OCD^8^ (1)
	7	36,103,748–37,353,138	19		height (2)
	11	21,262,535–21,390,555	98	Keratin-complex, IGFBP4	coat^9^ (1), hair^10^ (4)
	12	20,138,808–20,154,683	63	ZP1	insect (1), height (2)
	28	16,452,599–16,456,910	13		racing ^11^ (1)
**OLD**^**4**^	3	25,755,403	16		
	4	13,267,963–14,236,863	28	MYL7, CAMK2B, AEBP1, DBNL	
	6	25,827,163	31	GPC1	
	7	53,146,886–53,439,143	52	MTNR1B	gaits^12^ (1)
	9	17,420,422	16		
	10	33,830,402	25	HMGN3	
	11	18,231,316–18,256,680	52	SPATA32, WNT3, MYL4	height (1)
	15	47,471,803–47,772,350	5		tympany^13^ (3)
	16	52,972,887–53,092,742	15		
	17	16,506,700–16,546,991	18		
	19	4,177,482–4,193,516	12		
		52,321,709–53,872,140	32		height (2) sperm count (1)
	22	12,955,879–12,956,003	12		
		27,808,920–30,150,639	54	MYL9	

^1^ TRAK = Trakehner

^2^ HOL = Holsteiner

^3^ HAN = Hanoverian

^4^ OLD = Oldenburger

^5^ selection signature overlaps with QTL position, data downloaded from https://www.animalgenome.org/cgi-bin/QTLdb/index (accessed 4 February 2019)

^6^ height of withers

^7^ insect bite hypersensitivity

^8^ osteochondrosis dissecans

^9^ coat texture

^10^ hair density

^11^ racing ability

^12^ alternate gaits

^13^ guttural pouch tympany

### Enrichment analysis

The enrichment analysis based on genes located within across-breed iHS signatures (91 gene IDs recognised by DAVID out of 104 genes), identified the GO terms around nucleus, (tropo-) myosins, motor activity, insulin-like growth factor (IGF) and ATP binding to be enriched at p<0.05 (Table 6).

**Table 6:** Top 10 enriched pathways determined with DAVID from genes falling in across breed integrated Haplotype Score (iHS) selection signatures in four warmblood horse breeds.

**Term**	**%**^1^	**p**^2^	**Genes**	**FE**^3^	**BH**^4^
**nucleus (GO:0005634)**	23.08	9.37E-03	HECW1, FAM96A, TRIP4, ONECUT1, IKZF1, CSNK1G1, USP3, FIGNL1, PGAM2, RPS27L, STK17A, PSMA2, GABPB1, MAPK6, PPIB, GCK, ZPBP, POLM, LEO1, GNB5, IGFBP3	1.77	0.65
**motor activity (GO:0003774)**	3.30	1.35E-02	MYO5A, MYO1G, MYO5C	16.67	0.74
**myosin complex (GO:0016459)**	3.30	1.39E-02	MYO5A, MYO1G, MYO5C	16.42	0.55
**ATP binding (GO:0005524)**	15.38	1.47E-02	MYO5A, UBE2D4, DDX56, MAPK6, CSNK1G1, GCK, FIGNL1, MYO1G, CAMK2B, STK17A, DAPK2, ABCA13, ATP8B4, MYO5C	2.05	0.52
**exocytosis (GO:0006887)**	3.30	1.95E-02	MYO5A, MYO1G, YKT6	13.74	1.00
**ubiquitin-dependent protein catabolic process (GO:0006511)**	4.40	2.13E-02	PSMA2, USP8, USP3, USP50	6.68	0.97
**IGF I binding (GO:0031994)**	2.20	3.02E-02	IGFBP1, IGFBP3	64.45	0.64
**IGF II binding (GO:0031995)**	2.20	3.02E-02	IGFBP1, IGFBP3	64.45	0.64
**ruffle (GO:0001726)**	3.30	3.39E-02	MYO5A, DBNL, TLN2	10.22	0.73
**tropomyosin binding (GO:0005523)**	2.20	4.21E-02	TMOD2, TMOD3	46.04	0.65

^1^% = percentage of genes involved in pathway relative to all genes used for analysis

^2^ p = p-value

^3^ FE = fold enrichment

^4^ BH = Benjamini-Hochberg test

When taking genes falling into ROH stretches as input for the enrichment analysis (388 gene IDs recognised by DAVID out of 444 genes), the pathways IGF I and II binding were again detected, as well as IGF receptor signalling. Other nominally significant GO terms were intermediate filament, embryonic skeletal system morphogenesis and chondrocyte differentiation (Table 7)

**Table 7:** Top 10 enriched pathways determined with DAVID from genes falling in across breed Runs of Homozygosity (ROH) selection signatures in four warmblood horse breeds.

**Term**	**%**^1^	**p**^2^	**Genes**	**FE**^3^	**BH**^4^
**intermediate filament (GO:0005882)**	2.58	3.53E-07	KRT26, KRT25, NES, KRT28, KRT27, KRT12, KRT20, IFFO1, KRT23, KRT24	10.32	3.69E-05
**embryonic skeletal system morphogenesis (GO:0048704)**	1.80	1.04E-04	HOXB3, HOXB1, HOXB2, HOXB7, HOXB8, HOXB5, HOXB6	8.96	0.11
**structural molecule activity (GO:0005198)**	3.09	2.32E-04	KRT26, KRT25, NES, KRT28, EPB41L1, KRT27, KRT12, LMNA, KRT20, IFFO1, KRT23, KRT24	3.93	0.06
**anterior/posterior pattern specification (GO:0009952)**	2.06	1.41E-03	HOXB3, HOXB1, HOXB2, HOXB7, HOXB8, HOXB5, HOXB6, NEUROD1	4.74	0.53
**poly(A) RNA binding (GO:0044822)**	8.76	1.48E-03	FASTKD1, MTDH, PNPT1, HDGF, TRMT10A, WBP11, CCT3, MTIF2, POLR2B, MOV10, ARL6IP4, NQO1, FNDC3A, TOP2A, RBM12, RPS27A, ZCCHC8, NIP7, MEX3A, TBRG4, SSB, NOA1, ISG20L2, CASC3, DDX56, DDX55, PPIG, NOP2, EIF4E, HOXB6, POP1, SRP72, RBM39, WDR43	1.77	0.19
**IGF I binding (GO:0031994)**	0.77	5.29E-03	IGFBP1, IGFBP3, IGFBP4	25.35	0.40
**IGF II binding (GO:0031995)**	0.77	5.29E-03	IGFBP1, IGFBP3, IGFBP4	25.35	0.40
**chondrocyte differentiation (GO:0002062)**	1.29	6.39E-03	MEF2D, BMP2, WNT5B, GDF5, WNT2B	6.61	0.90
**hematopoietic progenitor cell differentiation (GO:0002244)**	1.55	1.12E-02	HOXB3, PTPN6, ARL11, GPATCH4, REST, TOP2A	4.41	0.95
**regulation of IGF receptor signaling pathway (GO:0043567)**	0.77	1.21E-02	IGFBP1, IGFBP3, IGFBP4	17.01	0.93

^1^% = percentage of genes involved in pathway relative to all genes used for analysis

^2^ p = p-value

^3^ FE = fold enrichment

^4^ BH = Benjamini-Hochberg test

When combining the genes from iHS and ROH signatures (461 unique IDs recognised out of 523 genes), the analysis for annotation clusters yielded four clusters with at least one individual GO term enriched at p< 0.05. The first cluster orbits around embryonic development, whereas the second one is based on IGF binding and cell growth. The third cluster focuses on cell proliferation, differentiation and fate, whereas the fourth cluster focusses on metabolism and glycolytic processes (Table 8). Here, embryonic skeletal system morphogenesis is the only biological process to pass the BH test.

**Table 8:** Top enriched annotation clusters determined with DAVID from genes falling in across breed integrated Haplotype Score (iHS) and Runs of Homozygosity (ROH) selection signatures in four warmblood horse breeds.

	Term	%^1^	P^2^	Genes	FE^3^	BH^4^
**Cluster 1** **ES^5^ = 1.96**	embryonic skeletal system morphogenesis (GO:0048704)	1.74	2.93E-05	HOXB3, HOXB1, HOXB2, HOXB7, HOXB8, HOXB5, HOXB6, GLI3	8.57	0.04
	anterior/posterior pattern specification (GO:0009952)	1.95	8.50E-04	HOXB3, HOXB1, HOXB2, HOXB7, HOXB8, HOXB5, HOXB6, NEUROD1, GLI3	4.46	0.42
	transcription factor activity, sequence-specific DNA binding (GO:0003700)	2.17	6.86E-01	CTBP1, IKZF3, HOXB2, HOXB7, IKZF1, HOXB8, HOXB6, MLXIP, GLI3, SCAND1	0.99	1.00
	sequence-specific DNA binding (GO:0043565)	1.30	8.36E-01	HOXB1, HOXB2, HOXB7, ETS1, HOXB6, HOXB13	0.85	1.00
**Cluster 2** **ES = 1.83**	IGF II binding (GO:0031995)	0.65	7.67E-03	IGFBP1, IGFBP3, IGFBP4	20.95	0.57
	IGF I binding (GO:0031994)	0.65	7.67E-03	IGFBP1, IGFBP3, IGFBP4	20.95	0.57
	regulation of IGF receptor signaling pathway (GO:0043567)	0.65	1.71E-02	IGFBP1, IGFBP3, IGFBP4	14.23	1.00
	regulation of cell growth (GO:0001558)	0.87	4.61E-02	CLSTN3, IGFBP1, IGFBP3, IGFBP4	4.92	1.00
**Cluster 3** **ES = 1.25**	chondrocyte differentiation (GO:0002062)	1.30	1.82E-03	SNX19, MEF2D, BMP2, WNT5B, GDF5, WNT2B	6.64	0.54
	cell fate commitment (GO:0045165)	0.87	9.87E-02	BMP2, WNT5B, ONECUT1, WNT2B	3.59	1.00
	Basal cell carcinoma	0.87	1.20E-01	BMP2, WNT5B, GLI3, WNT2B	3.28	0.94
	Hippo signaling pathway	1.08	4.74E-01	BMP2, WNT5B, GDF5, PPP1CB, WNT2B	1.41	0.96
**Cluster 4** **ES = 0.95**	glycolytic process (GO:0006096)	0.87	2.39E-02	TPI1, GCK, ENO2, PGAM2	6.33	0.99
	Glycolysis / Gluconeogenesis	1.08	5.40E-02	TPI1, GCK, ENO2, PGAM2, G6PC2	3.48	0.95
	Carbon metabolism	1.30	1.17E-01	HAO1, TPI1, GCK, ENO2, PGAM2, OGDH	2.30	0.95
	Biosynthesis of antibiotics	1.74	2.29E-01	HAO1, TPI1, GCK, ENO2, PGAM2, OGDH, PAICS, PPAT	1.60	0.94
	Biosynthesis of amino acids	0.65	5.24E-01	TPI1, ENO2, PGAM2	1.71	0.97

^1^% = percentage of genes involved in pathway relative to all genes used for analysis

^2^ p = p-value

^3^ FE = fold enrichment

^4^ BH = Benjamini-Hochberg test

^5^ ES = enrichment score

## Discussion

In this study we looked for signatures of selection within important equine warmblood horse breeds. In spite of their common relevance to sport horse breeding, their official current breeding focus differs with respect to sporting discipline. In addition, historically the four breeds Trakehner, Holsteiner, Hanoverian and Oldenburger underwent different breeding policies regarding pure and cross-breeding and divergent primary focus of utilization.

When seeking to evaluate and interpret this study’s results it should be kept in mind that the analysed sample set was preselected since only young stallions were included that had passed the studbooks’ first inspection and were sampled during the preselection’s health check. The sample is thus representative for the potential squad of sires of future generations and reflects the associations’ respective current breeding goals.

According to breeding documents, Trakehner and Holsteiner have most consistently pursued pure-breeding over the past century which is clearly reflected in the PCA clustering as well. The separation of Holsteiner from the other three breeds might also stem from their clear and relatively early focus on show-jumping. The sample set used in our study was already included in a study on the genomic prediction of breed assignment, in which an eigenvector analysis resulted in a very similar clustering [32]. Oldenburger and Hanoverian show a very similar clustering pattern in the PCA and also exhibit very similar iHS selection signatures. This concordance could originate both from shared breeding goals as well as the occasional common use of sires since the 1950s [48]. But the detected differences in the xpEHH-analysis show that both breeds have yet unique features that distinguish them from one another and might historical differences in breed formation. The xpEHH allows for pairwise breed comparisons and detects selection sites that are close to or have achieved fixation in one breed but remain diverse in another. Hence, it picks up signatures that are no longer detectable with the iHS or only result in weak signals.

Reduced local genetic variation is indicative of ongoing or past selection processes. This idea is implemented in the screening for ROH which refer to continuously homozygous segments in the genome. For the Trakehner horses, we found by far the highest number of breed-specific ROHs. On the one hand, the severe population bottleneck shortly after the Second World War is a possible cause for this phenomenon. On the other hand it is possible that simply more genomic sites have been under selective pressure compared to the other three breeds. The length of ROH can also shed light on the age of selection signatures and to what extend inbreeding is recent or dates further back. However, the average length of the ROHs within breed was not significantly different for any pairwise breed comparison, presumably because of the thresholds for SNP density and ROH assignment that were set for the ROH screening. A higher SNP resolution than used here would be necessary to obtain informative data on the precise length of the ROHs and thus indication on recent or historical selection events. However, results from within breed iHS analysis demonstrate the substantially divergent haplotype pattern and indicate distinct selection signatures in the Trakehner breed compared to the three others. This is in agreement with the reported divergent historical selection focus and breeding policy.

When searching for candidate genes under selection in our sample populations, we relied on overlaps of selection signatures with QTL, enriched pathways, functional candidacy and findings reported from other studies. The four breeds we investigated in this study mostly select for conformation, locomotion, athleticism and aptitude for one of the major disciplines show-jumping, dressage or eventing. Capability of reproduction, i.e. fertility, is also listed as a criterion by these studbooks [4, 9–12]. The results from the enrichment analyses in DAVID should be considered carefully. Although nominally significant (p<0.05), only two pathways were significantly enriched after a correction for multiple testing (Benjamini-Hochberg test).

Two ROH shared by at least a third of all individuals overlapped with QTL for hair density and coat texture (ECA 11). The enrichment analysis based on genes within ROH stretches showed an enrichment of the Gene Ontology (GO) term intermediate filament (GO:0005882). This was mostly driven by the keratin complex (ECA11). The xpEHH analysis between Hanoverian and the other three breeds also detected signatures spanning the keratin complex. Keratin is known to influence skin [49], hair [50] quality and is the major component of the equine hoof [51]. A missense variant in the coil1A domain of the *KRT 25* gene, which is located within our selection signatures, has previously been associated with the curly hair phenotype in horses [52]. In addition to the keratin complex, we suspect the gene KIT ligand (*KITLG*) to be under selective pressure. This gene has a well-documented effect on skin pigmentation and thereby coat colour in cattle [53] and pigs [53, 54] and is located within a ROH stretch on chromosome 28. Metzger et al. [26] also overserved homozygous segments around this locus and suggested *KITLG* as a selection target. Related to *KITLG* is *KIT* (tyrosine kinase receptor), which we found to be very close to a ROH signature on ECA 3 (75.8–76.3Mb) that also overlapped with a QTL for white markings [55]. *KIT* has been linked to dominant white syndrome in horses [56] as well as other coat colour phenotypes [57]. Throughout history, different coat colours have been favoured and targeted by selection in horses [58] and apparently this feature continues to be of relevance and under selection pressure [59].

Next to coat colour, size is a typical example of artificial selection in domestic animals [60]. Height of withers is a highly heritable [61] trait in horses that is easily measured and today a plethora of QTL is available for this trait [47]. We found QTL overlaps with ROH on ECA 3 and 8 [62] as well as overlaps with xpEHH selection signatures in all four breeds on multiple chromosomes (Trakehner: ECA 11 and 18; Holsteiner: ECA 7, 10 and 12; Hanoverian: ECA 7 and 12; Oldenburger: ECA 11 and 19).

Furthermore, a QTL for body weight [63] was located within a ROH stretch on ECA 18 in our analysis. The functional annotation of genes from ROH signatures resulted in an enrichment of the GO term regulation of cell growth (GO:0001558), which comprises the candidate genes *IGFBP* (insulin-like growth factor binding) *1*, *3* and *4*, which also feature in the biological process of regulation of *IGF* receptor signalling (GO:0043567) and the molecular function of *IGF I* and *II* binding (GO:0031994, GO:0031995).

Concluding from our results, we propose *IGF* binding proteins as new candidate genes for height of withers in horses, considering that *IGFBP4* has been associated with height in humans already [64]. *IGFBP1*, *3* and *4*, which we found in selection signatures (ECA 4 and 11), can bind to *IGF1* and *IGF2*, which are important for growth in early childhood [65].

An important factor for growth and body height in adolescence is organismal development in earlier stages of life. Genes located in ROH and iHS signatures were found enriched in an annotation cluster that revolved around prenatal development and specifically comprised the GO terms embryonic skeletal system morphogenesis (GO:0048704) and anterior/posterior pattern specification (GO:0009952). The *HOXB* gene cluster essentially underlying the enrichment of these pathways is very likely to be under selective pressure. *HOXB* genes are homeobox genes that are crucial for correct patterning of embryonic structures along the body axis, morphogenesis and nerval development [66]. Interestingly, the pathway for chondrocyte differentiation (GO:0002062) was part of annotation cluster 3 in our enrichment analysis (see Table 8). The gene *BMP2* (bone morphogenic protein 2), located within a ROH stretch on ECA 22, belongs to this pathway and has previously been associated with body size and development in sheep and goat [67, 68].

When looking at the biological background of athleticism, the two components (energy) metabolism and muscle functionality are of particular relevance [69, 70]. Our results give reason to assume that both components have been subject to selective pressure. To our knowledge, no association studies have been done in horses for metabolic traits or related traits. However, as mentioned before, the results from our enrichment analysis highlight *IGF I* and *II* binding and the regulation of *IGF* receptor signalling. Besides regulating growth, *IGF* binding proteins influence metabolism through the binding to *IGFs* and thereby manipulate glucose and insulin levels and are central players in diabetes, obesity and other metabolic diseases [71]. Both *IGFBP1* and *3* are related to insulin levels, fat accumulation (73), and have been linked to the metabolic syndrome (74), which also affects equids (75).

Naturally, many genes act in different pathways and may therefore be of special interest in breeding. Both *IGFBP4* and the gene *AEBP1* (adipocyte enhancer-binding protein 1) seem to play a double role in metabolism as well as muscle functioning. *AEBP1* falls within an across-breed iHS and a xpEHH signature in Oldenburger on ECA 4 and is reportedly involved in diet-induced obesity and energy homeostasis in mice, where it was upregulated in adipose tissue [72]. However, it is also a strong candidate for cardiac functioning and has been found to be highly expressed during the differentiation of smooth muscle cells of the aorta [73]. *IGFBP4* is a component involved in the canonical WNT-signaling pathway, which is necessary for cardiogenesis, where it exerts an inhibiting function [74].

Racing ability is one of the few performance traits analysed in association studies in horses. We found colocalisation of one QTL each with a ROH stretch on ECA 17 [75] and 18 [76] and an additional QTL for racing ability on ECA 28 [77] colocalised with a Hanoverian specific xpEHH signature. A ROH on ECA 22 spanned over *RALGAPA2* (Ral GTPase activating protein catalytic alpha subunit 2), which was already found in a selective sweep in Asian thoroughbreds and is reported to be associated with racing performance [78]. We assume that these regions harbour genes that contribute not only to racing ability but to sportiness in general.

Motor activity (GO:0003774) and myosin complex (GO:0016459), as well as tropomyosin binding (GO:0005523) stood out among the ten most significantly enriched GO terms in the analysis of genes localised in across-breed iHS signatures. Key players in these pathways were *MYO5A* (myosin VA), *MYO5C* (myosin VC), *MYO1G* (myosin IG), *TMOD2* and *TMOD3* (tropomodulin 2 & 3).

Sarcomeres are the contractile unit at the histological core of the muscle and comprise the two basic modules actin and myosin [79]. Many of the genes we found within or in proximity to selection sites encode for actin-binding proteins which already hints at their importance for sports performance orientated breeding. The genes *TPM1* (tropomyosin 1) and *TMOD2* & 3 were found in across breed iHS-signals on ECA 1. Both tropomyosin and tropomodulin are actin-binding and function as stabilizers for actin filaments. Mudry and colleagues [80] already reported that *TPM1* interacts with tropomodulin and aids to maintain and control actin filament length and is therefore important for cell structure and stability. The importance of *TPM1* for muscle functionality is further emphasized by findings in transgenic mice, where it was demonstrated that isoforms of *TPM1* govern muscle performance in cardiac and skeletal muscle [81].

While athletic performance is a trait that is clearly driven by artificial selection pressures, fertility is likely to be subject to natural selection processes. Low sperm quality in stallions correlates with pregnancy rates in mares [82] and it can be extrapolated that such stallions will generally produce less or no offspring. Analogously, mares with genetic predispositions for reproductive failure will produce less offspring or remain barren.

In contrast to height, fertility has much lower heritability [83, 84] and few GWAS have been performed for this feature. Unsurprisingly, we found only a single QTL overlap for sperm count [85] with a selection signature in our study and could not detect functional enrichment for a directly related pathway. Yet, there are functional candidate genes present in ROH and iHS selection signatures, such as *ZPBP1 & 2* (zona pellucida binding protein 1 & 2) and *SUN3* (Sad1 and UNC84 domain containing 3) on ECA 4 and 11. *SUN3* belongs to an interactive protein complex and is involved in sperm head formation in mammals [86] while the two known zona pellucida binding proteins *ZPBP1 & 2* play a crucial role in acrosome formation and morphological sperm development. The inactivation of either of the genes led to partial or full loss of fertility in mice [87] and mutations in *ZPBP1* were detected in infertile men, too [88]. The *ZPBP* is assigned to the GO term nucleus (GO:0005634), for which we found an enrichment based on genes localised in across breed iHS selection signatures.

Other genes possibly associated with male fertility are *THEGL* (testicular haploid expressed repeat spermatid protein like) and *TEX14* (testis expressed 14) in ROH stretches on ECA 3 and 11. Whilst *THEGL* has been found to be mainly expressed in testis and the ductus deferens in mice [89], *TEX14* plays a role in spermatogenesis [90]. Metzger et al. [26] proposed an additional gene as a selection candidate for male fertility in horses: *CFAP61* (Cilia and flagella associated protein 61) on ECA 22. Since we also detected a ROH across this gene, the results from our study support this hypothesis.

## Conclusion

This study revealed selection signatures in warmblood horses with a common current main breeding goal on athletic performance, but divergent historical breeding policy and selection focus. Despite breed specific differences, shared signals were found across the entire genome. Considering our findings and the analysis of annotated genes in regions under selective pressure, we conclude that candidate genes predominantly play a role in development and growth, metabolism, muscle development and functioning, as well as fertility. We suggest follow-up studies integrating comprehensively phenotyped warmblood sport horses with genomic information in order to validate whether the proposed candidate genes and genomic regions are indeed causal for variations in traits such as athletic performance.

## Supporting information

S1 Table Breed specific Runs of Homozygosity (ROH) in Holsteiner, Hanoverian, Oldenburger and Trakehner. ROH were shared by at least 33 percent of all individuals (N = 942) in the sample set.
(DOC)

S2 Table Breed specific significant integrated Haplotype Score (iHS) signals (-log_10_(p-value) ≥ 4.0) in Trakehner, Holsteiner, Hanoverian and Oldenburger. (DOC)
