# Supplementary material for: Selection signatures in four German warmblood horse breeds: Tracing breeding history in the modern sport horse
Source: PLoS One. 2019 Apr 25;14(4):e0215913. doi: 10.1371/journal.pone.0215913 (PMC6483353; doi:10.1371/journal.pone.0215913)
Supplement: S1 Table — ROH were shared by at least 33 percent of all individuals (N = 942) in the sample set. (DOC) [file pone.0215913.s001.doc]

|  | |  |  |  |  |  |  |  |  |  |  |  |  |  |
| --- | --- | --- | --- | --- | --- | --- | --- | --- | --- | --- | --- | --- | --- | --- |
| **Holsteiner** | | | | | | | | | | | | | | |
| **ECA** | **start position in basepairs** | | **end position in basepairs** | **start SNP** | **end SNP** | **length of ROH in basepairs** | **number SNPs in ROH** | **ECA** | **start position in basepairs** | **end position in basepairs** | **start SNP** | **end SNP** | **length of ROH in basepairs** | **number SNPs in ROH** |
| **1** | 33,006,515 | | 33,651,191 | BIEC2-14896 | BIEC2-15241 | 644,676 | 15 | **10** | 27,026,019 | 28,329,844 | BIEC2-111562 | BIEC2-111794 | 1,303,825 | 19 |
|  | 61,311,028 | | 61,826,124 | BIEC2-25335 | BIEC2-25439 | 515,096 | 16 | **11** | 6,027,707 | 6,790,047 | BIEC2-136187 | BIEC2-136339 | 762,340 | 20 |
| **2** | 96,475,507 | | 97,276,040 | BIEC2-500904 | BIEC2-501497 | 800,533 | 18 |  | 21,692,258 | 22,699,231 | BIEC2-143478 | BIEC2-143955 | 1,006,973 | 20 |
| **3** | 19,390,495 | | 20,068,977 | BIEC2-773771 | BIEC2-773848 | 678,482 | 20 |  | 24,038,863 | 24,990,015 | BIEC2-144586 | BIEC2-144877 | 951,152 | 16 |
|  | 23,079,499 | | 23,723,805 | BIEC2-774247 | BIEC2-774357 | 644,306 | 18 |  | 26,898,659 | 28,866,042 | BIEC2-145737 | BIEC2-147458 | 1,967,383 | 52 |
|  | 39,317,467 | | 40,650,253 | BIEC2-777987 | BIEC2-778563 | 1,332,786 | 20 |  | 32,231,193 | 34,080,306 | BIEC2-149160 | BIEC2-149980 | 1,849,113 | 42 |
|  | 57,214,428 | | 58,030,652 | BIEC2-783057 | BIEC2-783195 | 816,224 | 18 | **13** | 38,534,765 | 39,491,095 | BIEC2-235535 | BIEC2-235690 | 956,330 | 17 |
|  | 118,524,879 | | 119,456,949 | BIEC2-812036 | BIEC2-812208 | 932,070 | 38 |  | 41,914,786 | 42,556,495 | BIEC2-236127 | BIEC2-236256 | 641,709 | 23 |
| **4** | 19,079,166 | | 20,235,933 | BIEC2-852129 | BIEC2-852276 | 1,156,767 | 19 | **14** | 34,284,113 | 34,936,564 | BIEC2-254787 | BIEC2-255018 | 652,451 | 17 |
|  | 50,353,214 | | 51,482,212 | BIEC2-865189 | BIEC2-865698 | 1,128,998 | 26 | **15** | 43,450,922 | 44,082,288 | BIEC2-306081 | BIEC2-306462 | 631,366 | 15 |
|  | 52,575,697 | | 53,148,501 | BIEC2-865967 | BIEC2-866282 | 572,804 | 24 |  | 44,286,531 | 45,105,390 | BIEC2-306516 | BIEC2-306728 | 818,859 | 16 |
|  | 55,079,263 | | 55,680,047 | BIEC2-866929 | BIEC2-866993 | 600,784 | 16 |  | 58,240,235 | 59,448,498 | BIEC2-312257 | BIEC2-312377 | 1,208,263 | 16 |
| **5** | 41,810,883 | | 42,335,441 | BIEC2-907118 | BIEC2-907164 | 524,558 | 15 |  | 67,528,191 | 68,450,494 | BIEC2-316401 | BIEC2-316813 | 922,303 | 20 |
|  | 55,293,804 | | 56,274,553 | BIEC2-909788 | BIEC2-910331 | 980,749 | 28 | **16** | 30,485,436 | 31,463,061 | BIEC2-338172 | BIEC2-338734 | 977,625 | 18 |
| **6** | 24,261,234 | | 24,765,897 | BIEC2-945299 | BIEC2-945416 | 504,663 | 16 |  | 39,172,944 | 40,720,791 | BIEC2-341939 | BIEC2-342854 | 1,547,847 | 30 |
|  | 27,067,185 | | 28,058,021 | BIEC2-945760 | BIEC2-945846 | 990,836 | 23 |  | 47,364,808 | 47,958,319 | BIEC2-347054 | BIEC2-347340 | 593,511 | 19 |
|  | 28,856,527 | | 29,983,744 | BIEC2-946030 | BIEC2-946154 | 1,127,217 | 27 | **17** | 20,690,428 | 23,179,265 | BIEC2-373650 | BIEC2-373955 | 2,488,837 | 49 |
|  | 30,618,147 | | 31,231,313 | BIEC2-946284 | BIEC2-946421 | 613,166 | 19 | **18** | 39,150,093 | 39,786,446 | BIEC2-411251 | BIEC2-411383 | 636,353 | 18 |
|  | 34,014,417 | | 35,230,778 | BIEC2-947013 | BIEC2-947360 | 1,216,361 | 28 |  | 48,042,468 | 49,758,616 | BIEC2-412282 | BIEC2-412366 | 1,716,148 | 39 |
|  | 41,218,272 | | 42,816,830 | BIEC2-950410 | BIEC2-950900 | 1,598,558 | 28 |  | 58,873,993 | 60,310,646 | BIEC2-416307 | BIEC2-416468 | 1,436,653 | 33 |
|  | 63,589,518 | | 64,132,442 | BIEC2-1179230 | BIEC2-1179283 | 542,924 | 15 | **19** | 1,614,873 | 2,956,520 | BIEC2-422506 | BIEC2-423992 | 1,341,647 | 19 |
|  | 67,032,015 | | 68,434,085 | BIEC2-1180948 | BIEC2-1181090 | 1,402,070 | 32 | **21** | 14,484,223 | 15,040,604 | BIEC2-553662 | BIEC2-553948 | 556,381 | 16 |
| **7** | 36,663,831 | | 37,338,232 | BIEC2-995651 | BIEC2-996024 | 674,401 | 23 | **22** | 15,642,166 | 16,652,941 | BIEC2-583183 | BIEC2-583748 | 1,010,775 | 18 |
|  | 39,405,488 | | 40,906,478 | BIEC2-996506 | BIEC2-996658 | 1,500,990 | 37 |  | 24,107,087 | 24,818,381 | BIEC2-588905 | BIEC2-589172 | 711,294 | 20 |
| **8** | 22,113,249 | | 23,719,695 | BIEC2-1036498 | BIEC2-1037217 | 1,606,446 | 32 |  | 25,718,631 | 27,376,449 | BIEC2-589523 | BIEC2-590198 | 1,657,818 | 42 |
|  | 27,585,859 | | 30,790,573 | BIEC2-1039832 | BIEC2-1041556 | 3,204,714 | 54 | **25** | 26,318,531 | 26,942,120 | BIEC2-666776 | BIEC2-667279 | 623,589 | 18 |
|  | 92,759,642 | | 93,392,635 | BIEC2-1066463 | BIEC2-1066590 | 632,993 | 16 | **28** | 14,306,311 | 15,080,406 | BIEC2-730648 | BIEC2-730721 | 774,095 | 15 |
| **9** | 43,718,150 | | 44,540,142 | BIEC2-1090937 | BIEC2-1091258 | 821,992 | 20 |  | 45,594,055 | 46,121,975 | BIEC2-745495 | BIEC2-745550 | 527,920 | 17 |
|  | 45,662,085 | | 46,631,862 | BIEC2-1091531 | BIEC2-1091715 | 969,777 | 22 | **30** | 28,595,445 | 30,032,225 | BIEC2-828999 | BIEC2-829861 | 1,436,780 | 33 |
|  |  | |  |  |  |  |  |  |  |  |  |  |  |  |
| **Hanoverian** | | | | | | | | | | | | | | |
| **ECA** | | **start position in basepairs** | **end position in basepairs** | **start SNP** | **end SNP** | **length of ROH in basepairs** | **number SNPs in ROH** | **ECA** | **start position in basepairs** | **end position in basepairs** | **start SNP** | **end SNP** | **length of ROH in basepairs** | **number SNPs in ROH** |
| **1** | | 684,531 | 1,596,808 | BIEC2-98 | BIEC2-225 | 912,277 | 23 | **9** | 43,801,601 | 45,505,237 | BIEC2-1090979 | BIEC2-1091486 | 1,703,636 | 31 |
|  | | 21,809,520 | 23,066,972 | BIEC2-9966 | BIEC2-10963 | 1,257,452 | 33 | **11** | 24,038,863 | 24,990,015 | BIEC2-144586 | BIEC2-144877 | 951,152 | 16 |
|  | | 120,684,836 | 122,750,354 | BIEC2-52520 | BIEC2-52814 | 2,065,518 | 45 |  | 26,909,643 | 27,819,093 | BIEC2-145761 | BIEC2-146890 | 909,450 | 33 |
| **2** | | 100,347,967 | 101,659,052 | BIEC2-503102 | BIEC2-503409 | 1,311,085 | 35 | **14** | 62,662,526 | 63,226,417 | BIEC2-260799 | BIEC2-260854 | 563,891 | 16 |
| **3** | | 19,390,495 | 20,068,977 | BIEC2-773771 | BIEC2-773848 | 678,482 | 20 | **15** | 932,270 | 2,180,524 | BIEC2-278276 | BIEC2-279055 | 1,248,254 | 30 |
|  | | 22,704,683 | 23,723,805 | BIEC2-774181 | BIEC2-774357 | 1,019,122 | 26 |  | 44,286,531 | 45,105,390 | BIEC2-306516 | BIEC2-306728 | 818,859 | 16 |
|  | | 36,840,265 | 37,725,713 | BIEC2-777732 | BIEC2-777850 | 885,448 | 20 |  | 67,528,191 | 68,450,494 | BIEC2-316401 | BIEC2-316813 | 922,303 | 20 |
|  | | 75,713,271 | 76,496,451 | BIEC2-793471 | BIEC2-793815 | 783,180 | 22 | **16** | 34,059,162 | 35,013,749 | BIEC2-340127 | BIEC2-340465 | 954,587 | 23 |
|  | | 118,524,879 | 119,456,949 | BIEC2-812036 | BIEC2-812208 | 932,070 | 38 |  | 86,256,136 | 86,881,780 | BIEC2-365640 | BIEC2-365802 | 625,644 | 15 |
| **4** | | 15,120,680 | 17,575,687 | BIEC2-850770 | BIEC2-851384 | 2,455,007 | 51 | **17** | 20,762,784 | 21,823,211 | BIEC2-373659 | BIEC2-373775 | 1,060,427 | 17 |
|  | | 19,079,166 | 20,818,822 | BIEC2-852129 | BIEC2-852714 | 1,739,656 | 34 | **18** | 41,492,586 | 42,981,796 | BIEC2-411600 | BIEC2-411754 | 1,489,210 | 23 |
|  | | 24,221,403 | 26,434,219 | BIEC2-855686 | BIEC2-856750 | 2,212,816 | 43 |  | 48,042,468 | 50,094,655 | BIEC2-412282 | BIEC2-412389 | 2,052,187 | 44 |
|  | | 52,587,338 | 53,117,054 | BIEC2-865986 | BIEC2-866262 | 529,716 | 19 |  | 58,962,761 | 59,614,257 | BIEC2-416310 | BIEC2-416419 | 651,496 | 21 |
| **5** | | 41,546,213 | 42,575,094 | BIEC2-907076 | BIEC2-907178 | 1,028,881 | 27 | **22** | 4,359,148 | 4,944,734 | BIEC2-576849 | BIEC2-577192 | 585,586 | 15 |
| **6** | | 29,004,794 | 30,308,246 | BIEC2-946057 | BIEC2-946191 | 1,303,452 | 28 |  | 45,391,582 | 46,068,130 | BIEC2-600604 | BIEC2-600749 | 676,548 | 15 |
|  | | 34,084,026 | 34,849,024 | BIEC2-947027 | BIEC2-947135 | 764,998 | 20 | **25** | 25,922,684 | 27,010,162 | BIEC2-666479 | BIEC2-667349 | 1,087,478 | 24 |
|  | | 41,218,272 | 42,713,648 | BIEC2-950410 | BIEC2-950890 | 1,495,376 | 25 |  | 38,860,003 | 39,461,244 | BIEC2-674375 | BIEC2-674703 | 601,241 | 19 |
| **7** | | 39,405,488 | 41,489,510 | BIEC2-996506 | BIEC2-996688 | 2,084,022 | 47 | **28** | 14,015,630 | 14,681,697 | BIEC2-730532 | BIEC2-730674 | 666,067 | 21 |
| **8** | | 22,113,249 | 23,690,242 | BIEC2-1036498 | BIEC2-1037165 | 1,576,993 | 30 |  | 17,417,766 | 17,948,468 | BIEC2-732155 | BIEC2-732594 | 530,702 | 15 |
|  | | 35,977,142 | 37,944,491 | BIEC2-1044685 | BIEC2-1045512 | 1,967,349 | 48 |  |  |  |  |  |  |  |

| **Oldenburger** | | | | | | | | | | | | | |
| --- | --- | --- | --- | --- | --- | --- | --- | --- | --- | --- | --- | --- | --- |
| **ECA** | **start position in basepairs** | **end position in basepairs** | **start SNP** | **end SNP** | **length of ROH in basepairs** | **number SNPs in ROH** | **ECA** | **start position in basepairs** | **end position in basepairs** | **start SNP** | **end SNP** | **length of ROH in basepairs** | **number SNPs in ROH** |
| **1** | 684,531 | 1,596,808 | BIEC2-98 | BIEC2-225 | 912,277 | 23 | **8** | 22,113,249 | 23,690,242 | BIEC2-1036498 | BIEC2-1037165 | 1,576,993 | 30 |
|  | 15,419,110 | 16,093,214 | BIEC2-7424 | BIEC2-7563 | 674,104 | 18 |  | 36,329,323 | 37,791,469 | BIEC2-1044866 | BIEC2-1045440 | 1,462,146 | 25 |
|  | 21,809,520 | 23,066,972 | BIEC2-9966 | BIEC2-10963 | 1,257,452 | 33 | **9** | 43,718,150 | 45,505,237 | BIEC2-1090937 | BIEC2-1091486 | 1,787,087 | 35 |
| **2** | 100,347,967 | 100,961,025 | BIEC2-503102 | BIEC2-503162 | 613,058 | 19 | **11** | 24,038,863 | 25,885,998 | BIEC2-144586 | BIEC2-145234 | 1,847,135 | 34 |
| **3** | 19,511,517 | 20,068,977 | BIEC2-773784 | BIEC2-773848 | 557,460 | 16 |  | 26,909,643 | 27,819,093 | BIEC2-145761 | BIEC2-146890 | 909,450 | 33 |
|  | 22,704,683 | 23,723,805 | BIEC2-774181 | BIEC2-774357 | 1,019,122 | 26 |  | 32,322,500 | 33,535,388 | BIEC2-149173 | BIEC2-149790 | 1,212,888 | 27 |
|  | 39,317,467 | 40,650,253 | BIEC2-777987 | BIEC2-778563 | 1,332,786 | 20 | **14** | 62,722,596 | 63,332,961 | BIEC2-260802 | BIEC2-260866 | 610,365 | 17 |
|  | 75,713,271 | 76,496,451 | BIEC2-793471 | BIEC2-793815 | 783,180 | 22 | **15** | 44,286,531 | 45,105,390 | BIEC2-306516 | BIEC2-306728 | 818,859 | 16 |
|  | 118,524,879 | 119,456,949 | BIEC2-812036 | BIEC2-812208 | 932,070 | 38 |  | 67,528,191 | 68,378,301 | BIEC2-316401 | BIEC2-316789 | 850,110 | 18 |
| **4** | 15,120,680 | 17,465,064 | BIEC2-850770 | BIEC2-851325 | 2,344,384 | 43 | **18** | 48,760,138 | 50,094,655 | BIEC2-412320 | BIEC2-412389 | 1,334,517 | 30 |
|  | 19,079,166 | 20,235,933 | BIEC2-852129 | BIEC2-852276 | 1,156,767 | 19 |  | 53,908,214 | 54,462,617 | BIEC2-413536 | BIEC2-413944 | 554,403 | 16 |
|  | 22,288,213 | 23,579,902 | BIEC2-854320 | BIEC2-855456 | 1,291,689 | 21 |  | 58,962,761 | 59,614,257 | BIEC2-416310 | BIEC2-416419 | 651,496 | 21 |
|  | 49,486,277 | 50,765,212 | BIEC2-864840 | BIEC2-865296 | 1,278,935 | 25 |  | 69,343,747 | 69,931,904 | BIEC2-417718 | BIEC2-417796 | 588,157 | 15 |
|  | 52,587,338 | 53,117,054 | BIEC2-865986 | BIEC2-866262 | 529,716 | 19 | **22** | 999,404 | 1,668,631 | BIEC2-575173 | BIEC2-575385 | 669,227 | 18 |
| **5** | 48,519,041 | 49,603,095 | BIEC2-908049 | BIEC2-908650 | 1,084,054 | 21 |  | 4,359,148 | 4,944,734 | BIEC2-576849 | BIEC2-577192 | 585,586 | 15 |
| **6** | 29,004,794 | 30,308,246 | BIEC2-946057 | BIEC2-946191 | 1,303,452 | 28 |  | 15,147,832 | 16,561,219 | BIEC2-583117 | BIEC2-583687 | 1,413,387 | 27 |
|  | 34,084,026 | 34,849,024 | BIEC2-947027 | BIEC2-947135 | 764,998 | 20 | **24** | 42,072,495 | 42,751,348 | BIEC2-650750 | BIEC2-650822 | 678,853 | 17 |
|  | 41,218,272 | 42,713,648 | BIEC2-950410 | BIEC2-950890 | 1,495,376 | 25 | **25** | 26,318,531 | 26,942,120 | BIEC2-666776 | BIEC2-667279 | 623,589 | 18 |
| **7** | 39,405,488 | 45,559,043 | BIEC2-996506 | BIEC2-997272 | 6,153,555 | 78 | **28** | 17,417,766 | 17,948,468 | BIEC2-732155 | BIEC2-732594 | 530,702 | 15 |

| **Trakehner** | | | | | | | | | | | | | |
| --- | --- | --- | --- | --- | --- | --- | --- | --- | --- | --- | --- | --- | --- |
| **ECA** | **start position in basepairs** | **end position in basepairs** | **start SNP** | **end SNP** | **length of ROH in basepairs** | **number SNPs in ROH** | **ECA** | **start position in basepairs** | **end position in basepairs** | **start SNP** | **end SNP** | **length of ROH in basepairs** | **number SNPs in ROH** |
| **1** | 755,781 | 1,596,808 | BIEC2-103 | BIEC2-225 | 841,027 | 22 | **4** | 15,120,680 | 16,656,169 | BIEC2-850770 | BIEC2-851195 | 1,535,489 | 28 |
|  | 21,937,435 | 23,066,972 | BIEC2-10013 | BIEC2-10963 | 1,129,537 | 29 |  | 17,504,413 | 18,006,134 | BIEC2-851362 | BIEC2-851468 | 501,721 | 17 |
|  | 24,922,466 | 25,715,394 | BIEC2-11562 | BIEC2-11783 | 792,928 | 18 |  | 20,354,644 | 21,179,185 | BIEC2-852327 | BIEC2-853051 | 824,541 | 16 |
|  | 32,939,981 | 33,651,191 | BIEC2-14861 | BIEC2-15241 | 711,210 | 16 |  | 21,274,523 | 21,945,106 | BIEC2-853141 | BIEC2-853854 | 670,583 | 21 |
|  | 44,925,797 | 47,375,721 | BIEC2-20807 | BIEC2-21037 | 2,449,924 | 50 |  | 35,717,972 | 36,375,454 | BIEC2-858839 | BIEC2-858882 | 657,482 | 16 |
|  | 47,990,188 | 48,777,901 | BIEC2-21115 | BIEC2-21225 | 787,713 | 20 |  | 37,463,983 | 38,232,493 | BIEC2-858946 | BIEC2-859041 | 768,510 | 24 |
|  | 83,765,865 | 84,873,250 | BIEC2-36346 | BIEC2-36578 | 1,107,385 | 28 |  | 52,587,338 | 53,148,501 | BIEC2-865986 | BIEC2-866282 | 561,163 | 22 |
|  | 90,472,336 | 91,269,455 | BIEC2-37875 | BIEC2-38058 | 797,119 | 20 |  | 54,043,481 | 55,833,377 | BIEC2-866600 | BIEC2-866998 | 1,789,896 | 40 |
|  | 91,410,886 | 92,306,583 | BIEC2-38134 | BIEC2-38342 | 895,697 | 23 |  | 61,885,896 | 62,759,199 | BIEC2-868114 | BIEC2-868358 | 873,303 | 20 |
|  | 109,674,458 | 111,179,661 | BIEC2-46020 | BIEC2-46536 | 1,505,203 | 32 |  | 77,561,973 | 78,412,704 | BIEC2-870426 | BIEC2-870510 | 850,731 | 17 |
|  | 136,563,078 | 137,298,520 | BIEC2-59637 | BIEC2-60186 | 735,442 | 17 |  | 85,342,236 | 86,352,887 | BIEC2-872431 | BIEC2-872574 | 1,010,651 | 23 |
|  | 140,803,821 | 141,539,463 | BIEC2-61791 | BIEC2-61867 | 735,642 | 17 |  | 88,967,074 | 90,022,761 | BIEC2-873023 | BIEC2-873547 | 1,055,687 | 30 |
|  | 147,398,737 | 149,685,411 | BIEC2-63195 | BIEC2-64164 | 2,286,674 | 60 | **5** | 9,645,696 | 12,095,676 | BIEC2-891472 | BIEC2-892328 | 2,449,980 | 52 |
|  | 174,233,510 | 175,685,110 | BIEC2-83570 | BIEC2-84473 | 1,451,600 | 27 |  | 17,730,695 | 19,307,541 | BIEC2-896681 | BIEC2-897323 | 1,576,846 | 39 |
| **2** | 6,266,023 | 7,982,413 | BIEC2-454188 | BIEC2-454398 | 1,716,390 | 35 |  | 24,103,465 | 24,770,701 | BIEC2-898361 | BIEC2-898499 | 667,236 | 19 |
|  | 10,846,930 | 11,358,263 | BIEC2-455641 | BIEC2-456099 | 511,333 | 16 |  | 37,842,853 | 38,488,648 | BIEC2-905967 | BIEC2-906061 | 645,795 | 15 |
|  | 49,187,694 | 49,769,093 | BIEC2-477336 | BIEC2-477471 | 581,399 | 17 |  | 41,546,213 | 42,171,708 | BIEC2-907076 | BIEC2-907148 | 625,495 | 17 |
|  | 79,252,365 | 80,587,268 | BIEC2-491845 | BIEC2-492258 | 1,334,903 | 29 |  | 43,370,014 | 44,333,839 | BIEC2-907225 | BIEC2-907354 | 963,825 | 26 |
|  | 83,746,531 | 85,884,335 | BIEC2-493610 | BIEC2-494764 | 2,137,804 | 47 |  | 48,510,838 | 49,603,095 | BIEC2-908048 | BIEC2-908650 | 1,092,257 | 22 |
|  | 86,346,170 | 87,084,943 | BIEC2-494903 | BIEC2-495190 | 738,773 | 18 |  | 55,293,804 | 56,274,553 | BIEC2-909788 | BIEC2-910331 | 980,749 | 28 |
|  | 91,290,894 | 92,506,085 | BIEC2-497705 | BIEC2-498320 | 1,215,191 | 25 | **6** | 8,191,175 | 9,197,341 | BIEC2-938619 | BIEC2-938860 | 1,006,166 | 26 |
|  | 100,347,967 | 101,659,052 | BIEC2-503102 | BIEC2-503409 | 1,311,085 | 35 |  | 11,379,365 | 11,981,159 | BIEC2-939827 | BIEC2-940352 | 601,794 | 21 |
|  | 112,987,774 | 113,495,054 | BIEC2-507377 | BIEC2-507492 | 507,280 | 18 |  | 24,261,234 | 24,962,534 | BIEC2-945299 | BIEC2-945440 | 701,300 | 23 |
| **3** | 19,390,495 | 20,068,977 | BIEC2-773771 | BIEC2-773848 | 678,482 | 20 |  | 26,087,605 | 28,058,021 | BIEC2-945676 | BIEC2-945676 | 1,970,416 | 41 |
|  | 39,250,079 | 40,683,269 | BIEC2-777983 | BIEC2-778567 | 1,433,190 | 22 |  | 29,004,794 | 30,196,869 | BIEC2-946057 | BIEC2-946057 | 1,192,075 | 27 |
|  | 47,684,860 | 48,307,263 | BIEC2-779490 | BIEC2-779590 | 622,403 | 15 |  | 30,557,834 | 31,542,235 | BIEC2-946271 | BIEC2-946271 | 984,401 | 28 |
|  | 49,031,742 | 49,923,098 | BIEC2-779732 | BIEC2-779858 | 891,356 | 20 |  | 34,041,711 | 35,557,504 | BIEC2-947016 | BIEC2-947016 | 1,515,793 | 28 |
|  | 75,909,761 | 76,496,451 | BIEC2-793615 | BIEC2-793815 | 586,690 | 16 |  | 41,218,272 | 42,816,830 | BIEC2-950410 | BIEC2-950410 | 1,598,558 | 28 |
|  | 77,578,404 | 78,081,032 | BIEC2-794127 | BIEC2-794429 | 502,628 | 20 |  | 47,557,354 | 48,281,792 | BIEC2-953099 | BIEC2-953099 | 724,438 | 17 |
|  | 118,724,527 | 119,456,949 | BIEC2-812056 | BIEC2-812208 | 732,422 | 35 |  | 63,417,308 | 64,132,442 | BIEC2-1179221 | BIEC2-1179221 | 715,134 | 17 |
|  |  |  |  |  |  |  |  |  |  |  |  |  |  |
| **7** | 1,963,405 | 3,369,076 | BIEC2-974684 | BIEC2-974684 | 1,405,671 | 38 | **16** | 66,857,448 | 67,614,159 | BIEC2-356783 | BIEC2-356783 | 756,711 | 20 |
|  | 39,673,370 | 41,164,623 | BIEC2-996543 | BIEC2-996543 | 1,491,253 | 30 |  | 71,001,610 | 72,161,753 | BIEC2-358733 | BIEC2-358733 | 1,160,143 | 22 |
|  | 83,564,696 | 84,363,315 | BIEC2-1009163 | BIEC2-1009163 | 798,619 | 23 | **17** | 7,377,084 | 8,328,734 | BIEC2-367804 | BIEC2-367804 | 951,650 | 16 |
|  | 93,871,120 | 94,992,399 | BIEC2-1017317 | BIEC2-1017317 | 1,121,279 | 27 |  | 20,762,784 | 21,823,211 | BIEC2-373659 | BIEC2-373659 | 1,060,427 | 17 |
| **8** | 27,865,163 | 30,729,188 | BIEC2-1039998 | BIEC2-1039998 | 2,864,025 | 52 |  | 22,683,951 | 23,485,614 | BIEC2-373877 | BIEC2-373877 | 801,663 | 20 |
|  | 35,977,142 | 38,463,436 | BIEC2-1044685 | BIEC2-1044685 | 2,486,294 | 59 |  | 26,733,895 | 27,385,825 | BIEC2-374483 | BIEC2-374483 | 651,930 | 18 |
|  | 39,653,906 | 40,402,407 | BIEC2-1046000 | BIEC2-1046000 | 748,501 | 18 |  | 27,693,738 | 28,523,193 | BIEC2-374698 | BIEC2-374698 | 829,455 | 16 |
|  | 40,835,755 | 41,866,538 | BIEC2-1046141 | BIEC2-1046141 | 1,030,783 | 23 |  | 46,536,494 | 47,210,393 | BIEC2-377259 | BIEC2-377259 | 673,899 | 16 |
|  | 55,974,524 | 56,555,498 | BIEC2-1053403 | BIEC2-1053403 | 580,974 | 15 |  | 57,138,276 | 57,962,518 | BIEC2-378565 | BIEC2-378565 | 824,242 | 16 |
|  | 63,912,872 | 64,445,288 | BIEC2-1058193 | BIEC2-1058193 | 532,416 | 15 |  | 73,574,890 | 74,140,286 | BIEC2-383940 | BIEC2-383940 | 565,396 | 20 |
| **9** | 4,153,419 | 5,935,244 | BIEC2-1070230 | BIEC2-1070230 | 1,781,825 | 36 |  | 79,880,795 | 80,701,963 | BIEC2-387527 | BIEC2-387527 | 821,168 | 25 |
| **10** | 27,026,019 | 28,329,844 | BIEC2-111562 | BIEC2-111562 | 1,303,825 | 19 | **18** | 9,856,035 | 11,743,497 | BIEC2-399593 | BIEC2-399593 | 1,887,462 | 27 |
| **11** | 32,186,583 | 33,449,725 | BIEC2-113281 | BIEC2-113281 | 1,263,142 | 21 |  | 20,614,378 | 21,601,340 | BIEC2-408459 | BIEC2-408459 | 986,962 | 25 |
|  | 77,011,124 | 77,851,298 | BIEC2-131922 | BIEC2-131922 | 840,174 | 18 |  | 23,481,561 | 24,529,189 | BIEC2-409039 | BIEC2-409172 | 1,047,628 | 20 |
|  | 14,115,164 | 14,620,504 | BIEC2-140385 | BIEC2-140385 | 505,340 | 17 |  | 30,239,261 | 31,447,118 | BIEC2-410026 | BIEC2-410112 | 1,207,857 | 25 |
|  | 21,490,673 | 24,044,680 | BIEC2-143292 | BIEC2-143292 | 2,554,007 | 60 |  | 32,821,448 | 33,392,131 | BIEC2-410309 | BIEC2-410405 | 570,683 | 20 |
|  | 25,158,631 | 25,885,998 | BIEC2-144966 | BIEC2-144966 | 727,367 | 15 |  | 37,625,509 | 38,345,198 | BIEC2-411057 | BIEC2-411142 | 719,689 | 15 |
|  | 26,158,220 | 28,649,035 | BIEC2-145349 | BIEC2-145349 | 2,490,815 | 52 |  | 41,492,586 | 42,981,796 | BIEC2-411600 | BIEC2-411754 | 1,489,210 | 23 |
|  | 32,322,500 | 34,204,408 | BIEC2-149173 | BIEC2-149173 | 1,881,908 | 42 |  | 47,108,529 | 50,189,654 | BIEC2-412179 | BIEC2-412399 | 3,081,125 | 62 |
|  | 36,069,308 | 36,978,846 | BIEC2-150383 | BIEC2-150383 | 909,538 | 18 |  | 68,721,497 | 69,931,904 | BIEC2-417640 | BIEC2-417796 | 1,210,407 | 31 |
| **12** | 29,032,022 | 30,451,343 | BIEC2-197916 | BIEC2-197916 | 1,419,321 | 31 | **19** | 14,506,238 | 15,072,254 | BIEC2-430118 | BIEC2-430208 | 566,016 | 18 |
| **13** | 38,387,299 | 41,502,477 | BIEC2-235502 | BIEC2-235502 | 3,115,178 | 70 |  | 17,338,313 | 17,991,705 | BIEC2-430594 | BIEC2-430721 | 653,392 | 19 |
| **14** | 41,649,129 | 43,564,128 | BIEC2-257155 | BIEC2-257155 | 1,914,999 | 47 |  | 19,076,234 | 19,856,434 | BIEC2-430900 | BIEC2-431021 | 780,200 | 20 |
|  | 61,655,636 | 62,467,767 | BIEC2-260581 | BIEC2-260581 | 812,131 | 17 |  | 34,325,308 | 35,014,501 | BIEC2-436412 | BIEC2-436734 | 689,193 | 15 |
|  | 62,662,526 | 63,332,975 | BIEC2-260799 | BIEC2-260799 | 670,449 | 19 |  | 37,551,287 | 38,734,966 | BIEC2-437740 | BIEC2-438348 | 1,183,679 | 33 |
|  | 68,974,256 | 70,422,247 | BIEC2-263349 | BIEC2-263349 | 1,447,991 | 36 |  | 56,468,957 | 57,205,127 | BIEC2-447082 | BIEC2-447705 | 736,170 | 15 |
| **15** | 31,961,275 | 32,545,364 | BIEC2-301405 | BIEC2-301405 | 584,089 | 21 | **20** | 9,569,614 | 10,736,947 | BIEC2-517206 | BIEC2-517798 | 1,167,333 | 23 |
|  | 44,286,531 | 44,842,635 | BIEC2-306516 | BIEC2-306516 | 556,104 | 15 |  | 42,047,525 | 44,389,829 | BIEC2-532574 | BIEC2-533450 | 2,342,304 | 51 |
|  | 67,764,699 | 68,378,301 | BIEC2-316527 | BIEC2-316527 | 613,602 | 15 | **21** | 4,197,576 | 5,809,552 | BIEC2-547743 | BIEC2-548307 | 1,611,976 | 30 |
|  | 71,041,540 | 71,956,116 | BIEC2-318224 | BIEC2-318224 | 914,576 | 23 |  | 24,242,194 | 25,316,610 | BIEC2-557516 | BIEC2-558231 | 1,074,416 | 20 |
|  | 75,841,270 | 76,436,521 | BIEC2-320461 | BIEC2-320461 | 595,251 | 18 |  | 26,216,980 | 26,994,829 | BIEC2-558692 | BIEC2-559140 | 777,849 | 22 |
|  | 79,263,331 | 80,085,648 | BIEC2-321571 | BIEC2-321571 | 822,317 | 21 |  | 55,271,059 | 57,654,847 | BIEC2-573754 | BIEC2-574130 | 2,383,788 | 54 |
|  | 30,746,944 | 31,720,566 | BIEC2-338256 | BIEC2-338256 | 973,622 | 19 |  |  |  |  |  |  |  |
|  |  |  |  |  |  |  |  |  |  |  |  |  |  |
| **22** | 999,404 | 1,668,631 | BIEC2-575173 | BIEC2-575385 | 669,227 | 18 |  |  |  |  |  |  |  |
|  | 4,138,064 | 4,944,734 | BIEC2-576687 | BIEC2-577192 | 806,670 | 18 |  |  |  |  |  |  |  |
|  | 14,837,927 | 16,561,219 | BIEC2-583018 | BIEC2-583687 | 1,723,292 | 33 |  |  |  |  |  |  |  |
|  | 17,487,160 | 18,272,412 | BIEC2-584230 | BIEC2-584230 | 785,252 | 19 |  |  |  |  |  |  |  |
|  | 40,114,705 | 40,664,988 | BIEC2-597290 | BIEC2-597290 | 550,283 | 16 |  |  |  |  |  |  |  |
|  | 40,765,245 | 41,505,016 | BIEC2-597867 | BIEC2-597867 | 739,771 | 17 |  |  |  |  |  |  |  |
|  | 44,016,700 | 45,095,728 | BIEC2-600273 | BIEC2-600273 | 1,079,028 | 23 |  |  |  |  |  |  |  |
|  | 46,699,016 | 47,712,171 | BIEC2-600887 | BIEC2-600887 | 1,013,155 | 33 |  |  |  |  |  |  |  |
|  | 49,340,717 | 49,870,421 | BIEC2-601624 | BIEC2-601624 | 529,704 | 15 |  |  |  |  |  |  |  |
| **23** | 10,307,107 | 11,208,836 | BIEC2-611893 | BIEC2-611893 | 901,729 | 23 |  |  |  |  |  |  |  |
| **24** | 12,770,201 | 14,058,563 | BIEC2-633765 | BIEC2-633765 | 1,288,362 | 33 |  |  |  |  |  |  |  |
|  | 41,552,201 | 42,751,348 | BIEC2-650621 | BIEC2-650621 | 1,199,147 | 21 |  |  |  |  |  |  |  |
| **25** | 765,420 | 1,390,245 | BIEC2-653399 | BIEC2-653399 | 624,825 | 15 |  |  |  |  |  |  |  |
|  | 1,877,375 | 2,456,894 | BIEC2-653802 | BIEC2-653802 | 579,519 | 18 |  |  |  |  |  |  |  |
|  | 26,318,531 | 27,125,754 | BIEC2-666776 | BIEC2-666776 | 807,223 | 22 |  |  |  |  |  |  |  |
| **26** | 22,954,773 | 25,949,044 | BIEC2-690523 | BIEC2-690523 | 2,994,271 | 74 |  |  |  |  |  |  |  |
|  | 26,924,886 | 27,923,174 | BIEC2-691927 | BIEC2-691927 | 998,288 | 19 |  |  |  |  |  |  |  |
| **27** | 6,306,812 | 8,257,142 | BIEC2-701550 | BIEC2-701550 | 1,950,330 | 38 |  |  |  |  |  |  |  |
|  | 13,011,581 | 14,319,372 | BIEC2-705352 | BIEC2-705352 | 1,307,791 | 29 |  |  |  |  |  |  |  |
|  | 16,895,805 | 17,601,828 | BIEC2-707269 | BIEC2-707269 | 706,023 | 15 |  |  |  |  |  |  |  |
|  | 36,789,720 | 38,069,413 | BIEC2-720851 | BIEC2-720851 | 1,279,693 | 38 |  |  |  |  |  |  |  |
| **28** | 14,306,311 | 15,080,406 | BIEC2-730648 | BIEC2-730648 | 774,095 | 15 |  |  |  |  |  |  |  |
|  | 45,228,144 | 46,121,975 | BIEC2-745470 | BIEC2-745470 | 893,831 | 20 |  |  |  |  |  |  |  |
| **30** | 28,783,979 | 29,494,895 | BIEC2-829139 | BIEC2-829139 | 710,916 | 20 |  |  |  |  |  |  |  |
