# Supplementary material for: Selection signatures in four German warmblood horse breeds: Tracing breeding history in the modern sport horse
Source: PLoS One. 2019 Apr 25;14(4):e0215913. doi: 10.1371/journal.pone.0215913 (PMC6483353; doi:10.1371/journal.pone.0215913)
Supplement: S2 Table — (DOC) [file pone.0215913.s002.doc]

|  |  |  |  |  |  |  |  |  |
| --- | --- | --- | --- | --- | --- | --- | --- | --- |
| **Trakehner** | | | |  | **Holsteiner** | | | |
| **ECA** | **SNP-position base pairs** | **iHS** | **-log_10_(p-value)** |  | **ECA** | **SNP-position base pairs** | **iHS** | **-log_10_(p-value)** |
| 1 | 35,768,895 | 4.55 | 5.27 |  | 17 | 23,169,975 | 3.91 | 4.04 |
| 4 | 39,143,318 | 4.22 | 4.62 |  | 17 | 23,175,862 | 3.91 | 4.04 |
| 4 | 39,143,502 | 4.22 | 4.62 |  | 17 | 23,176,167 | 3.91 | 4.04 |
| 4 | 39,143,508 | 4.22 | 4.62 |  |  |  |  |  |
| 12 | 29,746,984 | 4.09 | 4.36 |  |  |  |  |  |
| 18 | 49,758,616 | 3.98 | 4.16 |  |  |  |  |  |
|  |  |  |  |  |  |  |  |  |
| **Hanoverian** | | | |  | **Oldenburger** | | | |
| **ECA** | **SNP-position base pairs** | **iHS** | **-log_10_(p-value)** |  | **ECA** | **SNP-position base pairs** | **iHS** | **-log_10_(p-value)** |
| 1 | 121,759,945 | 3.98 | 4.16 |  | 1 | 128,778,389 | -3.92 | 4.05 |
| 1 | 128,778,389 | -4.11 | 4.39 |  | 1 | 128,801,482 | 3.94 | 4.09 |
| 1 | 128,801,482 | 4.16 | 4.50 |  | 1 | 128,829,558 | -4.01 | 4.22 |
| 1 | 128,829,558 | -4.18 | 4.54 |  | 1 | 137,759,895 | 4.05 | 4.29 |
| 1 | 130,177,232 | 4.04 | 4.28 |  | 1 | 138,481,053 | 3.97 | 4.15 |
| 1 | 138,718,660 | 4.29 | 4.75 |  | 1 | 139,162,818 | 4.20 | 4.58 |
| 1 | 138,722,881 | 3.98 | 4.16 |  | 1 | 139,266,776 | 4.09 | 4.37 |
| 1 | 139,083,018 | 4.04 | 4.28 |  | 4 | 13,965,265 | 4.53 | 5.22 |
| 1 | 139,162,818 | 4.19 | 4.56 |  | 4 | 14,310,388 | 3.99 | 4.17 |
| 1 | 139,266,776 | 3.96 | 4.12 |  | 4 | 16,091,738 | 4.01 | 4.22 |
| 4 | 13,965,265 | 4.29 | 4.74 |  | 4 | 16,091,813 | 4.01 | 4.22 |
| 4 | 16,091,738 | 4.47 | 5.11 |  | 4 | 17,504,413 | -3.96 | 4.12 |
| 4 | 16,091,813 | 4.47 | 5.11 |  | 4 | 18,006,134 | 4.01 | 4.22 |
| 4 | 16,095,704 | 4.12 | 4.42 |  | 7 | 39,673,370 | 4.69 | 5.55 |
| 4 | 17,455,561 | 4.28 | 4.74 |  | 7 | 39,673,699 | -3.94 | 4.09 |
| 4 | 18,448,170 | 4.00 | 4.19 |  |  |  |  |  |
| 4 | 18,448,561 | 4.00 | 4.19 |  |  |  |  |  |
| 4 | 19,079,166 | -4.11 | 4.41 |  |  |  |  |  |
| 4 | 19,190,415 | -4.16 | 4.51 |  |  |  |  |  |
| 4 | 19,190,566 | -4.19 | 4.55 |  |  |  |  |  |
| 4 | 20,181,845 | 4.11 | 4.41 |  |  |  |  |  |
| 4 | 20,228,735 | -4.07 | 4.33 |  |  |  |  |  |
